# Exploring Structure–Activity Relationships of Niclosamide-Based Colistin Potentiators in Colistin-Resistant Gram-Negative Bacteria

**DOI:** 10.3390/antibiotics13010043

**Published:** 2024-01-03

**Authors:** Liam Berry, Quinn Neale, Rajat Arora, Danyel Ramirez, Marc Brizuela, Ronald Domalaon, Gilbert Arthur, Frank Schweizer

**Affiliations:** 1 Department of Chemistry, University of Manitoba, Winnipeg, MB R3T 2N2, Canada; berryl3@myumanitoba.ca (L.B.); nealeq@myumanitoba.ca (Q.N.); arorar5@myumanitoba.ca (R.A.); ramiredm@myumanitoba.ca (D.R.); marc.brizuela@umanitoba.ca (M.B.);; 2 Department of Biochemistry and Medical Genetics, University of Manitoba, Winnipeg, MB R3E 3N4, Canada; gilbert.arthur@umanitoba.ca; 3 Department of Medical Microbiology and Infectious Diseases, University of Manitoba, Winnipeg, MB R3E 0J9, Canada

**Keywords:** colistin, niclosamide, antibiotic, adjuvant, Gram-negative bacteria

## Abstract

Colistin is primarily used as a last resort antibiotic against highly resistant Gram-negative bacteria (GNB). Rising rates of colistin resistance, however, may limit future use of this agent. The anthelmintic drug niclosamide has been shown to enhance colistin activity in combination therapy, but a detailed structure–activity relationship (SAR) for niclosamide against GNB has yet to be studied. A series of niclosamide analogs were synthesized to perform an SAR, leading to the discovery of a lead compound that displayed comparable colistin-potentiating activity to niclosamide with reduced cytotoxicity. Overall, this work provides important insights into synthetic strategies for the future development of new niclosamide derivatives and demonstrates that toxicity to mammalian cells can be reduced while maintaining colistin potentiation.

## 1. Introduction

The widespread and ever-increasing threat of antimicrobial-resistant (AMR) “superbugs” presents a significant danger to the healthcare system and to the entire world [1,2]. Indeed, a substantial increase in infections caused by AMR Gram-negative bacteria (GNB) has led to the increase in usage of last resort antibiotics such as colistin. However, rising rates of colistin resistance have resulted in bacterial infections that are resistant to all currently used antibiotics, leading to the deaths of several patients [3]. If no new treatments for these challenging infections are developed, humanity may be left with no effective agents with which to eradicate these “superbugs”. 

A significant barrier to the development of new antibiotics is the challenging and costly drug approval process [4]. This combined with limited return on investment for treating rare AMR infections has stagnated the development of new antibiotics [5]. One method to overcome this significant challenge is to repurpose existing FDA-approved drugs towards the fight against AMR bacteria [6]. The already established toxicity and pharmacokinetic profiles of these drugs may streamline and potentially truncate the development and approval process for such agents, rendering a quick pathway for their much needed use in the clinic. An example of such is the anthelmintic drug niclosamide approved for the treatment of tapeworm infections, which has been shown to exhibit antibacterial activity against Gram-positive bacteria [7], inhibit quorum sensing in Gram-negative bacteria [8], and able to synergize with colistin against GNB [9] to combat the development of colistin resistance.

Despite being a promising candidate for repurposing as a drug against AMR infections, niclosamide has notable limitations. In particular, niclosamide has an extremely poor bioavailability primarily due to its low solubility [10]. Toxicity to mammalian cells has also been observed which has spurred interest in repurposing niclosamide as an anticancer agent [11] but is a significant hindrance for developing it as a selective and safe antibacterial agent. In order to further develop niclosamide as an adjuvant capable of augmenting colistin and overcome colistin resistance, new derivatives must be synthesized, and their structure–activity relationships need to be studied. Several publications have reported new niclosamide analogs for combating colistin resistance [12], as well as a detailed mechanistic understanding of niclosamide in combination with colistin [13].

**Figure 1:**
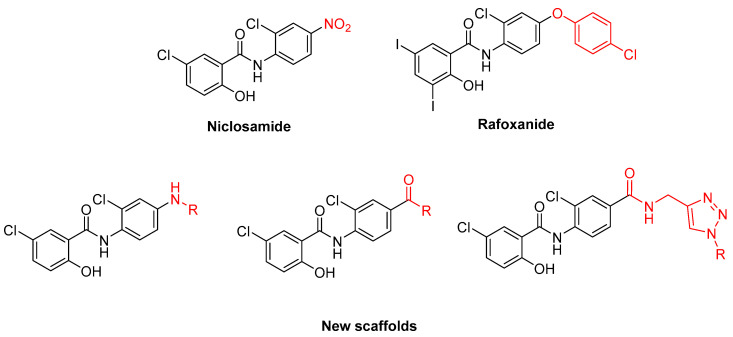
Structures of niclosamide, rafoxanide, and proposed new scaffolds. The initial site of modification is highlighted.

The aim of this study is to synthesize derivatives of niclosamide with the goal of reducing toxicity to human cells while retaining synergy with colistin. Niclosamide is composed of a 5-chlorosalicylic acid ring linked to a 2-chloro-4-nitroaniline ring via an amide bond. The nitro group has been demonstrated to contribute to genotoxicity [14], as well as serving as a target for bacterial nitroreductases resulting in resistance to niclosamide [13]. These factors, in addition to reports that structurally similar salicylanilides such as rafoxanide (Figure 1) which lack the nitro group can also synergize with colistin [15], led to us targeting this site for most of our modifications. Our initial synthetic goal was the replacement of the nitro group with an amide moiety, which would allow rapid synthesis of a small library of amide analogs incorporating various functional groups. We also explored attaching azide and alkyne functional groups to the niclosamide scaffold with the goal of producing a small library of triazole-containing compounds via copper-assisted azide-alkyne cycloaddition (CuACC) [16].

In this work, it was found that the nitro group in niclosamide could be replaced with a methyl ester, an azide, or (to a lesser extent) a primary amine while retaining synergy with colistin against colistin-resistant GNB. We also show that replacement of the nitro group can result in new derivatives of niclosamide with lower cytotoxicity against human cells. Overall, this work provides important insights towards future development and optimization of niclosamide for overcoming colistin resistance.

## 2. Results and Discussion

### 2.1. Chemistry

**Scheme 1:**
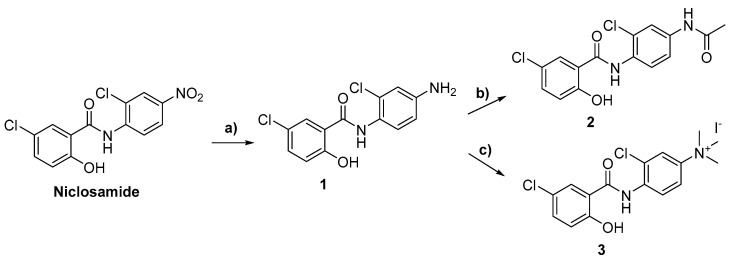
Synthesis of amine derivatives of niclosamide (a) Zn, NH_4_Cl, MeOH/THF, rt (b) CH_3_COCl, DCM, rt (c) CH_3_I, 2,6 lutidine, DMF, rt.

The nitro group on niclosamide was chosen as an initial site for modification. We initially sought to synthesize two series of amide analogs, one of which linked the amide to the salicylanilide scaffold via the nitrogen and one that was linked via the carbonyl group. The first series of derivatives resulted from treating niclosamide with zinc dust in the presence of NH_4_Cl, yielding primary amine derivative **1**. As an initial proof of concept, the amine was further acylated and alkylated to produce compounds **2** and **3** (Scheme 1). 

**Scheme 2:**
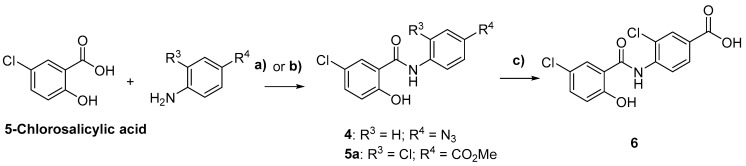
Synthesis of azide, ester, and carboxylic acid derivatives of niclosamide. (a) EDC, THF, rt (b) PCl_3_, xylenes, reflux (c) LiOH (aq), THF/MeOH, rt.

**Scheme 3:**
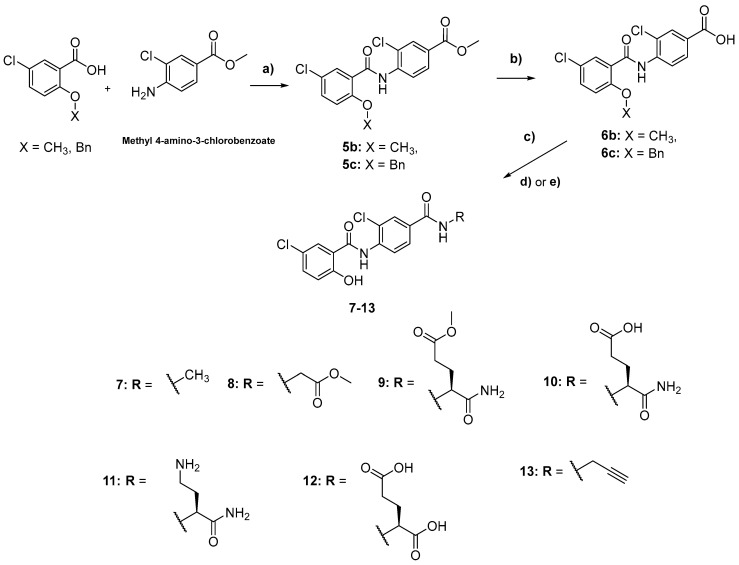
Synthesis of amide derivatives of niclosamide (a) PCl_3_/xylenes, reflux; (b) LiOH (aq), THF/MeOH; (c) TBTU, NMM, DMF, NH_2_-R; (d) BBr_3_, DCM, 0 °C; (e) H_2_ Pd/C, MeOH/THF.

The other series of amide containing compounds was synthesized by coupling commercially available 5-chlorosalisylic acid with various aniline derivatives. A methyl ester derivative **5a** was synthesized via PCl_3_ coupling. Ester hydrolysis with LiOH produced carboxylic acid analogue **6** which was intended to serve as a scaffold to produce a small library of amide analogues (Scheme 2 and Scheme 3). 

Initial attempts to derivatize acid **6** via amide coupling proved unsuccessful with analysis revealing the phenol in niclosamide posing synthetic challenges. Two phenol protecting group strategies were developed to overcome this challenge: a methyl ether protecting group that could be deprotected using BBr_3_ and a benzyl protecting group that could be deprotected with catalytic hydrogenolysis. The role of the phenol functional group was also explored using intermediate **5b**, analogous to **5a** but with a methyl ether protected phenol. These protecting group strategies enabled the development of amide derivatives **7**–**12** which allowed for the introduction of a variety of functional groups (Scheme 3). 

**Scheme 4:**
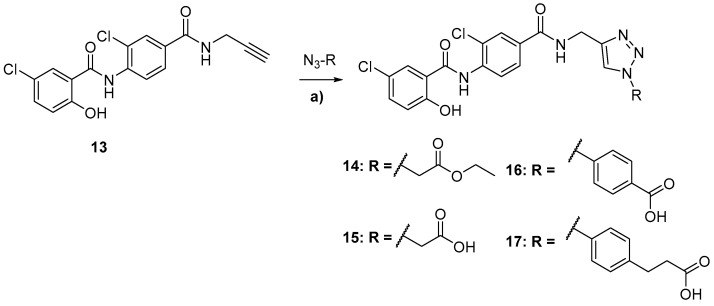
Synthesis of triazole derivatives of niclosamide (a) CuI⋅P(OEt)_3_, *i*Pr_2_Net, DMF, rt, 2 h.

An azide derivative **4** was also prepared by reacting 5-chlorosalisylic acid with 4-azidoaniline in the presence of 1-ethyl-3-(3-dimethylaminopropyl)carbodiimide (EDC). Attempts to synthesize an ethynylaniline derivative with an alkyne at the same position as the azide group proved unsuccessful, so an alternative strategy to install the alkyne group was utilized. Compound **13**, a propargylamide derivative, was coupled to various azide-containing fragments via copper-assisted azide-alkyne cycloaddition (CuAAC) [16] in Scheme 4 to explore the effects of a triazole moiety in compounds **14**–**17**. 

**Scheme 5:**
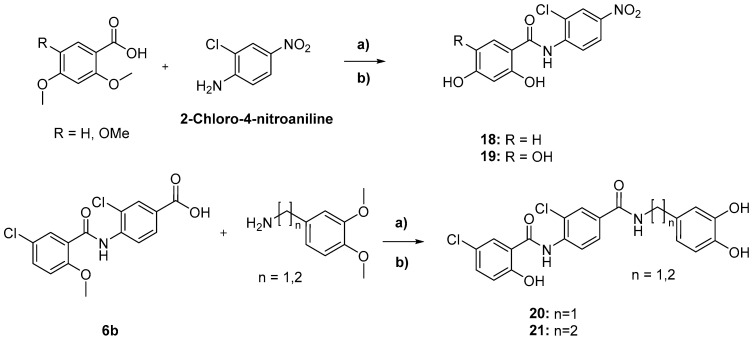
Synthesis of polyphenol derivatives of niclosamide. (a) PCl_3_, xylenes, reflux (b) BBr_3_, DCM, 0 °C.

Lastly, a series of polyphenol derivatives were synthesized (Scheme 5). Since the phenol group on niclosamide was previously shown to be essential for its activity as a protonophore [17] and thus its synergy with colistin [13], we wanted to explore the effect of installing additional phenol groups. The salicylic acid ring of niclosamide was modified with additional hydroxyl groups (**18**, **19**) and the previously described amide coupling method was used to produce polyphenol analogs **20** and **21**. 

### 2.2. Biological Evaluations

#### 2.2.1. Initial Screen for Synergy with Colistin and SAR

The initial screen of our study aimed to evaluate the ability of compounds **1**–**21** to synergize with colistin. For the initial screen, two colistin-resistant clinical isolates of *Klebsiella pneumoniae* (KP113250 and KP113254) and *Escherichia coli* (EC94393 and EC94474) were chosen. The two *K. pneumoniae* strains were chosen as they are highly colistin-resistant with minimum inhibitory concentrations (MIC) for colistin of 256 μg/mL. The two *E. coli* strains were selected as they were known to harbor the *mcr-1* gene [18] which confers colistin resistance via phosphoethanolamine transferase [19]. Colistin had MICs of 8 μg/mL in EC94394 and 16 μg/mL in EC94474. 

To ensure any biological activity seen was due to colistin potentiation and not to any innate activity of the newly synthesized compounds, we first screened all compounds for standalone antibacterial activity against these four strains (Table S1). The lowest concentration of each compound able to inhibit bacterial growth was determined to be the MIC. Similar to niclosamide, all compounds displayed poor antibacterial activity with MIC values of ≥128 μg/mL and were then further evaluated for synergy with colistin. 

To perform the initial screen, we used a fixed concentration of 4 μM of compound and determined the MIC of colistin in combination against each strain. As a comparison, niclosamide was shown to drastically enhance the antibacterial activity of colistin in all four strains, consistent with previous reports [9]. At 4 μM, niclosamide was able to lower the MIC of colistin by 512-fold against KP113250, 1024-fold against KP113254, and 32-fold against both *E. coli* strains. The prominent colistin MIC reduction by niclosamide provided a baseline of potentiating activity to which the new derivatives could be compared. At first, we investigated colistin potentiation for compounds **1**–**3** at 4 μM (Table 1)**.** We observed that the conversion of the nitro group to an amine moiety (**1**) resulted in comparable potentiation of colistin in *E. coli* strains. However, reduced potentiation was observed against *K. pneumoniae*. Further modifying the amine with acylation (**2**) or alkylation (**3**) led to a complete loss of colistin-potentiating activity against both bacteria (Table 1). 

We next replaced the nitro group on niclosamide with an azide (**4**), a methyl ester (**5a**), a carboxylic acid (**6**), various amide moieties (**7**–**12**), and triazole fragments (**14**–**17**) (Table 2). We observed that the azide and methyl ester derivatives retained synergy with colistin against all strains tested, indicating that these modifications are compatible with the colistin potentiation of niclosamide. Compound **4** displayed prominent potentiating activity against the two *E. coli* strains, reducing the MIC of colistin to 0.016 μg/mL against EC94393 (a 16-fold higher reduction compared to niclosamide). As **4** also contains hydrogen at R^1^ instead of a chlorine atom, it appears that the chlorine at this position is not necessary for synergy with colistin. Compound **5a**, in combination with colistin, displayed a comparable synergy profile relative to niclosamide in all tested strains. Interestingly, compound **5b**, which differs from **5a** only by a methylated phenol group, lost all synergy with colistin (Table S2). This demonstrates the necessity of the phenol in niclosamide’s ability to potentiate colistin. Unfortunately, further modification of the methyl ester moiety by converting it to a carboxylic acid (**6**) or an amide (**7**–**12**) completely abolished all synergy with colistin. The triazole analogs **14**–**17** were not able to potentiate colistin in all tested strains. Nonetheless, these findings demonstrated that the nitro group on niclosamide can be replaced with a variety of substituents while still retaining synergy with colistin. 

Given the demonstrated importance of the phenol moiety within the salicylanilide scaffold, we wondered how the installation of additional phenol groups, as in compounds **18**–**21**, would affect niclosamide’s ability to potentiate colistin (Table 3). We observed that the addition of even one additional phenol group on the salicylic acid ring (**18**) resulted in a complete loss of synergy with colistin. The amide-containing polyphenol compounds **20** and **21** were also inactive. These results suggest that while the phenol group is necessary, the addition of multiple phenol groups reduces colistin-potentiating activity.

Overall, the results of this SAR led to us conclude that the nitro group in niclosamide can be replaced with an azide, a methyl ester, and (to a lesser extent) an amine while still retaining synergy with colistin against GNB. We also show that further modification of the amine or ester groups abolishes synergistic activity. The phenol group was shown to be necessary for synergy with colistin as methylation resulted in complete loss of colistin potentiation (compound **5b**, Table S2). 

**Figure 2:**
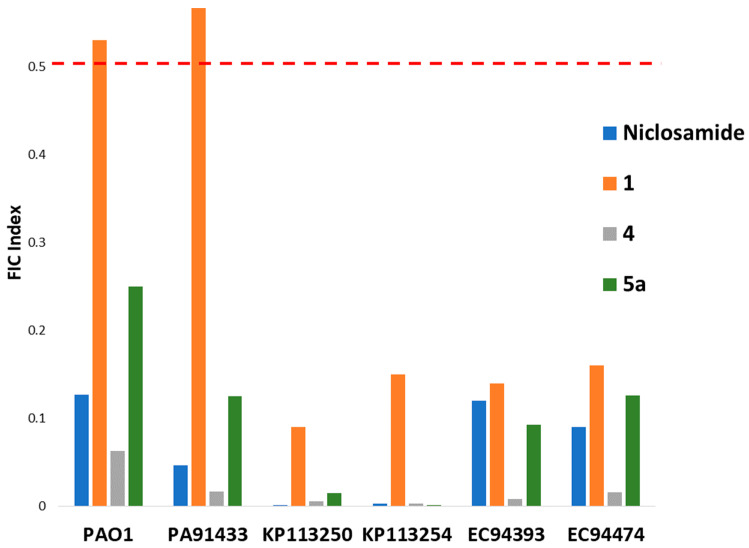
Synergy comparison between compounds **1**, **5a,** or **4** and niclosamide. PA = *Pseudomonas aeruginosa*, KP = *Klebsiella pneumoniae,* EC = *Escherichia coli*. Red dotted line denotes FICI of 0.5. FICI values were obtained at a concentration of 4 μM for each compound.

Finally, we compared the colistin potentiation of hit compounds **1**, **4** and **5a** to niclosamide in a panel of colistin-resistant and colistin-susceptible GNB using the checkerboard assay (Figure 2). The fractional inhibitory concentration index (FICI) for each combination was determined to assess interactions between the two components. FICI values of ≤0.5, 0.5 < x ≤ 4, and >4 were interpreted as synergistic, additive, and antagonistic interactions, respectively. We observed that compound **4** was overall superior to niclosamide (lowest FICI value), especially in *E. coli.* Compound **5a** was comparable to niclosamide, while compound **1** showed synergy with colistin only in *E. coli* and *K. pneumoniae* but not in *Pseudomonas aeruginosa.* Despite the promising colistin-potentiating activity of **4**, the high synthetic cost as well as concerns over the stability of the aryl azide led to us selecting the methyl ester compound **5a** for further screening against clinical isolates of *P. aeruginosa* and *Acinetobacter baumannii*.

#### 2.2.2. Synergy between **5a** and Colistin against *P. aeruginosa* and *A. baumannii*

We further evaluated the therapeutic potential of lead compound **5a** in combination with colistin against a panel of multidrug-resistant (MDR) clinical isolates of *P. aeruginosa* and *A. baumannii* (Table 4 and Table 5). Compound **5a** was able to synergize with colistin in 8 out of 10 strains of *P. aeruginosa.* Synergy was only not observed in PA259 and PA260, which were already highly susceptible to colistin (MIC of 0.25 μg/mL). Analog **5a** was able to drastically reduce colistin’s MIC against all colistin-resistant isolates (PA91433, PA101243, PA262 and PA101885). The combination of **5a** and colistin against PA101243 was of particular note, with 1 μg/mL of **5a** lowering the MIC of colistin from 1024 μg/mL to 0.5 μg/mL, a 2048-fold reduction. In all strains tested, **5a** was able to lower the MIC of colistin to 0.5 μg/mL or less (Table 4). 

Similar synergistic activity was observed for *A. baumannii* (Table 5). Synergy was observed in four out of five isolates, LAC-4 (colistin MIC of 0.25 μg/mL) being the only exception. Compound **5a** was able to lower the MIC of colistin to 0.5 μg/mL or below in all strains tested, including against highly resistant AB027 (colistin MIC of 1024 μg/mL). 

Overall, these results show that a combination of colistin and **5a** is effective against *K. pneumonia, E. coli, P. aeruginosa* and *A. baumannii.* Replacing the nitro group of niclosamide with a methyl ester led to the development of a new derivative that synergized with colistin in nearly all clinical isolates tested, and restored colistin activity against every colistin-resistant isolate. 

#### 2.2.3. Cytotoxicity of **1** and **5a** against Eukaryotic Cells

**Figure 3:**
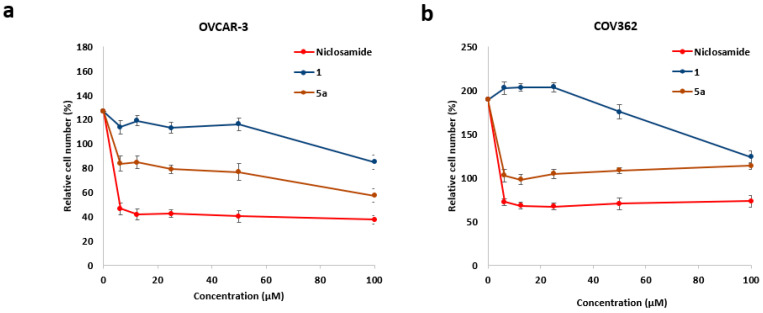
Effect of niclosamide, compound **1** and compound **5a** (0–100 µM) on ovarian cancer cell lines (**a**) OVCAR-3 and (**b**) COV362 cells. Cell numbers from representative wells were determined on day 0 before the addition of the compound. After 48 h incubation (day 2), cells were counted again to determine the increase in cell number over day 0 cell count. The overall increase in cell number for control after 48 h incubation was found to be around 26% and 89% relative to the initial cell number (day 0) for OVCAR-3 and COV362 cells, respectively. Each concentration of the drug tested is represented as a percent of that cell growth relative to the initial cell number. The results represent the mean ± standard deviation of two independent experiments with four wells for each concentration.

Toxicity against eukaryotic cells is a major challenge for repurposing niclosamide as an antibacterial agent. To test whether the modification of the nitro group may be a potential avenue for reducing toxicity, the toxic effects of compounds **1** and **5a** were investigated on two ovarian cancer cell lines OVCAR-3 and COV362, and compared to niclosamide which has been shown to possess potent antitumor effects against ovarian cancer cells [20]. The results of experiments with the three compounds using the CyQuant assay [21] are displayed in Figure 3. After 48 h incubation, the cell number for the controls increased by about 26% for OVCAR-3 and 89% for COV362 relative to the initial cell number (day 0).

Incubation with 6–100 μM niclosamide after 48 h showed a toxic effect on both the cell lines, killing around 60% (OVCAR-3) and 30% (COV362) of the initial cell number before drug treatment. Incubation of cells with compound **5a** at 6–50 μM killed only 20% of OVCAR-3 cells while it had a cytostatic effect (no significant change in initial cell number) in the same concentration range in COV362 cells. On the other hand, compound **1** at 6–50 μM showed slight inhibition of proliferation (around 13%) in OVCAR-3 cells relative to the control whereas under the same incubation conditions at 6–25 µM in COV362 cells, it stimulated proliferation and increased the cell number by 14% more than the control. Thus, in both OVCAR-3 and COV362 cells, incubation with compound **1** resulted in cell numbers higher than the initial cell number indicating a non-cytotoxic effect in the range of 6–25 μM.

Compound **5a** and compound **1** at 100 μM killed 43% and 15% of OVCAR-3 cells, respectively. At the same concentration, in COV362 cells, compound **5a** and compound **1** showed 75% and 66% inhibition of proliferation relative to the control but the cell numbers were still higher than the initial cell number indicating an antiproliferative rather than a cytotoxic effect. Taken together, the results demonstrate that compounds **1** and **5a** are less cytotoxic than niclosamide at all concentrations tested. Since the results of the microbiological studies demonstrate that compound **5a** was able to synergize with colistin to eradicate colistin-resistant GNB strains tested at concentrations of 1μg/mL (~0.3 μM), these results are very promising as an initial demonstration of reduced cytotoxicity. Overall, these results show that the replacement of the nitro group in niclosamide is a viable strategy to reduce toxicity against eukaryotic cells while retaining synergy with colistin.

## 3. Materials and Methods

### 3.1. Materials

Reagents and solvents were purchased from commercially available suppliers such as Sigma-Aldrich (St Louis, MO, USA), AK Scientific (Union City, CA, USA), Fisher Scientific (Waltham, MA, USA), and Bachem (Bubendorf, Switzerland) and were used without further purification. Air- and moisture-sensitive reactions were performed with dry solvents under inert nitrogen atmosphere. The reaction progress was monitored using thin-layer chromatography (TLC) plates visualized with UV light. Column chromatography was performed to purify the compounds using SiliaFlash P60 (40–63 μm) 60 Å silica gel from Silicycle (Quebec City, QC, Canada). The chemical structures of all final products used for biological testing were characterized by nuclear magnetic resonance spectroscopy (^1^H NMR and ^13^C NMR) on a Bruker AMX NMR 300 MHz and 500 MHz spectrometer (Bruker, Billerica, MA, USA). Electrospray ionization mass spectrometry (ESI-MS) data were obtained using a Varian 320-MS LC/MS (Agilent, Santa Clara, CA, USA).

### 3.2. Synthetic Methods

#### General Synthetic Procedures

General procedure A: Benzanilide coupling using PCl_3_

The appropriate benzoic acid derivative (1 equiv.) and aniline derivative (1 equiv.) were dissolved in dry xylene (2 mL per mmol of reactants) and heated to reflux. PCl_3_ (0.4 equiv.) was added dropwise upon which a precipitate formed. Solution was refluxed for 4 h. The mixture was cooled to rt and filtered, after which the precipitate was washed with ethyl acetate. 

General procedure B: LiOH Ester hydrolysis

The appropriate carboxylic acid was dissolved in a THF (5 mL per mmol) and MeOH (5 mL per mmol) mixture. A solution of 2M LiOH in water (10 mL per mmol) was added and the reaction was stirred at rt for 2 h. The reaction was neutralized with concentrated HCl at 0 °C upon which the product precipitated, was filtered and washed with water (3 × 10 mL). 

General procedure C: Amide coupling using TBTU

The appropriate carboxylic acid (1 equiv.), *N*-methylmorpholine (3 equiv.), and O-(Benzotriazol-1-yl)-N,N,N′,N′-tetramethyluronium tetrafluoroborate (TBTU) (1.1 equiv.) were dissolved in anhydrous DMF (3 mL per mmol of carboxylic acid) and stirred at rt for 15 min under N_2_. The appropriate amine (1.2 equiv.) was added and the reaction was stirred for 4 h at rt. Solution was concentrated in vacuo and the residue was collected in DCM, washed with 1M HCl (×3), saturated NaHCO_3_ (×3) and brine. The organic layer was concentrated and the product was purified using flash chromatography (0–10% MeOH in DCM).

General procedure D: Methoxy deprotection

The appropriate methoxy protected intermediate (1 equiv.) was dissolved in anhydrous DCM at 0 °C. A 1M solution of BBr_3_ in DCM (1.5 equiv. BBr_3_ per methoxy group) was added dropwise and the reaction was slowly warmed to rt. Solution was stirred overnight. The reaction was cooled to 0 °C and quenched with the careful addition of MeOH (5 mL per mmol) followed by stirring for 15 min. The reaction mixture was concentrated in vacuo and the product was purified using flash chromatography (0–10% MeOH in DCM).

General procedure E: Benzyl deprotection

The appropriate benzyl protected intermediate (1 equiv.) was dissolved in MeOH (3 mL per mmol) and THF (1 mL per mmol) Palladium on carbon (0.1 equiv.) was then added followed by H_2_ gas (balloon). The solution was stirred for 2 h at rt, filtered over celite and concentrated in vacuo. The product was purified using flash chromatography (0–10% MeOH in DCM).

General procedure F: Copper-assisted azide-alkyne cycloaddition (CuAAC)

The appropriate azide (1 equiv.) and alkyne (1 equiv.) and CuI·P(OEt)_3_ (0.3 equiv.) were dissolved in anhydrous DMF (3 mL) and treated with iPr_2_NEt (2 equiv.) The reaction was stirred under N_2_ at rt for 2 h. The reaction mixture was concentrated under vacuum, dissolved in water and extracted with DCM (× 3) The organic layers were combined, dried over anhydrous Na_2_SO_4_, and concentrated in vacuo. The product was purified using flash chromatography (0–10% MeOH in DCM).

(**1**) *N*-(4-amino-2-chlorophenyl)-5-chloro-2-hydroxybenzamide

Niclosamide (1.435 g, 4.38 mmol) was suspended in 25 mL of 1:1 MeOH/THF, followed by the addition of 10 mL of saturated NH_4_Cl (aq). Zinc dust (3 g, 45.89 mmol) was slowly added into the solution at 0 °C. The reaction was warmed to RT and stirred for 4 h, after which TLC indicated that the starting material was completely consumed. The solution was filtered over celite to remove excess zinc and the resulting filtrate was concentrated in vacuo. The crude residue was dissolved in 30 mL ethyl acetate and washed with water (3 × 10 mL) and brine (3 × 5 mL). The organic layer was concentrated and purified by flash chromatography (0–10% MeOH in DCM) yielding 649.5 mg (2.19 mmol, 49.9%) of compound **1** as a pale yellow solid. ^1^H NMR (500 MHz, Methanol-*d*_4_) δ 8.64 (d, *J* = 8.9 Hz, 1H), 8.02 (d, *J* = 2.7 Hz, 1H), 7.56 (d, *J* = 2.5 Hz, 1H), 7.41–7.37 (m, 2H), 6.97 (d, *J* = 8.8 Hz, 1H). ^13^C NMR (126 MHz, Methanol-*d*_4_) δ 163.49, 155.16, 136.00, 133.39, 130.08, 126.86, 124.82, 124.55, 123.72, 123.19, 122.03, 119.42, 118.14. ESI-MS: M/Z [M+H]^+^ 297.0193. 

(**2**) *N*-(4-acetamido-2-chlorophenyl)-5-chloro-2-hydroxybenzamide

Compound **1** (88 mg, 0.3 mmol) was dissolved in 3 mL DCM followed by the addition of Et_3_N (125 µL, 0.9 mmol) and acetyl chloride (25 µL, 0.36 mmol). Solution was stirred at RT overnight and concentrated in vacuo. The crude residue was dissolved in 5 mL ethyl acetate and washed with saturated NaHCO_3_ (3 × 5 mL) and brine (3 × 3 mL). The organic layer was dried over anhydrous Na_2_SO_4_, concentrated and purified by flash chromatography (0–10% MeOH in DCM) yielding 86.6 mg (0.25 mmol, 84.8%) **2** off-white powder.^1^H NMR (500 MHz, DMSO-*d*_6_) δ 10.88 (s, 1H), 10.76 (s, 1H), 8.24 (d, *J* = 8.9 Hz, 1H), 8.01 (d, *J* = 2.3 Hz, 1H), 7.97 (d, *J* = 2.8 Hz, 1H), 7.58 (dd, *J* = 9.0, 2.4 Hz, 1H), 7.47 (dd, *J* = 8.8, 2.8 Hz, 1H), 7.32 (d, *J* = 8.8 Hz, 1H), 2.07 (s, 3H). ^13^C NMR (126 MHz, DMSO- *d*_6_) δ 169.14, 163.02, 156.37, 137.21, 133.54, 130.32, 129.98, 123.99, 123.57, 123.51, 119.99, 119.75, 119.59, 118.44, 24.37. ESI-MS: M/Z [M+H]^+^ 339.0312.

(**3**) 3-chloro-4-(5-chloro-2-hydroxybenzamido)-*N*,*N*,*N*-trimethylanilinium

Compound **1** (250 mg, 0.85 mmol) was dissolved in 5 mL DMF. 2,6 lutidine (186 µL, 1.6 mmol) was added followed by iodomethane (211 µL, 3.4 mmol). Solution was stirred at RT overnight and 20 mL ethyl acetate was added to facilitate precipitation. Product was washed on-filter with ethyl acetate (3 × 5 mL), acetone (3 × 5 mL) and DCM (3 × 5 mL) yielding 142 mg (0.41 mmol, 49%) **3** as a dark brown solid. ^1^H NMR (500 MHz, DMSO-*d*_6_) δ 12.38 (s, 1H), 11.17 (s, 1H), 8.67 (d, *J* = 8.7 Hz, 1H), 8.22 (d, *J* = 2.2 Hz, 1H), 8.05–7.92 (m, 2H), 7.49 (d, *J* = 8.75 Hz, 1H), 7.06 (d, *J* = 8.4 Hz, 1H), 3.56 (s, 9H). ^13^C NMR (126 MHz, DMSO-*d*_6_) δ 166.14, 155.73, 143.25, 136.31, 132.87, 130.21, 129.98, 123.99, 123.57, 123.62, 119.87, 118.92, 118.01, 56.87. ESI-MS: M/Z [M+H]^+^ 339.0661.

(**4**) *N*-(4-azidophenyl)-5-chloro-2-hydroxybenzamide

5-Chlorosalicylic acid (45 mg, 0.293 mmol) and EDC (112 mg, 0.586 mmol) were dissolved in tetrahydrofuran (2 mL) at room temperature and stirred for 15 min.

4-Azidoaniline (51 mg, 0.299 mmol) was added and the reaction mixture was stirred at rt for 5 h over which time the solution gradually turned from clear to yellow. The reaction was concentrated under vacuo and dissolved in 12 mL ethyl acetate, washed with 1 M HCl (3 × 5 mL), saturated sodium bicarbonate solution (3 × 5 mL) and brine (3 × 5 mL), dried over anhydrous Na_2_SO_4_ and concentrated in vacuo. The crude material was purified using flash chromatography using DCM as a solvent system to afford 4 as a brown solid (61 mg, 73%) ^1^H NMR (500 MHz, DMSO-*d*_6_) δ 11.81 (s, 1H), 10.47 (s, 1H), 7.93 (d, *J* = 2.6 Hz, 1H), 7.76 (dd, *J* = 9.0, 2.9 Hz, 2H), 7.48 (dd, *J* = 8.8, 2.6 Hz, 1H), 7.16–7.13 (m, 2H), 7.02 (d, *J* = 8.8 Hz, 1H). ^13^C NMR (126 MHz, DMSO-*d*_6_) δ 177.32, 165.42, 157.41, 143.04, 135.53, 133.49, 128.77, 122.63, 122.60, 119.67, 119.63.

(**5a**) methyl 3-chloro-4-(5-chloro-2-hydroxybenzamido)benzoate

Compound **5a** was synthesized following general procedure A starting from 5-Chlorosalicylic acid (928 mg, 5.38 mmol) and methyl 4-amino-3-chlorobenzoate (1 g, 5.38 mmol) and purified by flash chromatography (0–10% MeOH in DCM) resulting in 748 mg (2.2 mmol, 41%) **5a** as a white solid. ^1^H NMR (500 MHz, DMSO-*d*_6_) δ 12.37 (s, 1H), 11.14 (s, 1H), 8.67 (d, *J* = 8.7 Hz, 1H), 8.01 (d, *J* = 2.0 Hz, 1H), 7.98–7.92 (m, 2H), 7.50 (dd, *J* = 8.7, 2.8 Hz, 1H), 7.08 (d, *J* = 8.8 Hz, 1H), 3.85 (s, 3H). ^13^C NMR (126 MHz, DMSO-*d*_6_) δ 165.15, 162.85, 155.55, 139.79, 134.15, 130.46, 130.35, 129.55, 125.99, 124.14, 122.81, 121.53, 120.09, 119.53, 52.75. ESI-MS: M/Z [M+H]^+^ 340.0148.

(**5b**) methyl 3-chloro-4-[(5-chloro-2-methoxybenzoyl)amino]benzoate

Compound **5b** was synthesized following general procedure A starting from 5-Chloro-2-methoxybenzoic Acid (558 mg, 3 mmol) and methyl 4-amino-3-chlorobenzoate (516 g, 3 mmol) resulting in 840 mg (2.4 mmol, 82%) of **5b** as a white solid. ^1^H NMR (300 MHz, Chloroform-*d*) δ 10.80 (s, 1H), 8.81 (d, *J* = 8.8 Hz, 1H), 8.29 (d, *J* = 2.8 Hz, 1H), 8.12 (d, *J* = 2.0 Hz, 1H), 8.05–7.94 (m, 1H), 7.50 (dd, *J* = 8.9, 2.8 Hz, 1H), 7.02 (d, *J* = 8.9 Hz, 1H), 4.12 (s, 3H), 3.94 (s, 3H). ^13^C NMR (75 MHz, Chloroform-*d*) δ 165.69, 162.19, 155.90, 139.56, 133.55, 132.37, 130.48, 129.49, 127.19, 125.90, 122.47, 120.84, 113.11, 56.77, 52.33. ESI-MS: M/Z [M+H]^+^ 354.0292.

(**5c**) methyl 4-{[2-(benzyloxy)-5-chlorobenzoyl]amino}-3-chlorobenzoate

Compound **5a** (100 mg, 0.3 mmol) was dissolved in 3 mL anhydrous DMF under N_2_ at 0 °C. NaH (13 mg, 0.32 mmol) as a 60% dispersion in mineral oil was added and the solution was stirred at 0 °C for 15 min. Benzyl bromide (39 µL, 0.32 mmol) was added dropwise and the solution was warmed to RT and stirred for 4 h. The reaction was quenched with 10 mL 1M HCl (aq) at 0 °C which caused the product to precipitate. The precipitate was filtered and washed with 3 × 5 mL hexanes yielding 107 mg of **5c** as a pale-yellow solid (0.25 mmol, 83%) which was used without further purification.

(**6**) 3-chloro-4-(5-chloro-2-hydroxybenzamido)benzoic acid

Compound **6** was obtained using general procedure B starting from **5a** (708 mg, 2.1 mmol) in 98% (667 mg) yield as a white powder. ^1^H NMR (300 MHz, DMSO-*d*_6_) δ 11.38 (s, 1H), 8.61 (d, *J* = 8.6 Hz, 1H), 7.95 (d, *J* = 1.9 Hz, 1H), 7.91–7.86 (m, 2H), 7.53–7.45 (m, 2H). ^13^C NMR (75 MHz, DMSO-*d*_6_) δ 166.09, 163.06, 156.65, 139.29, 133.87, 130.44, 130.13, 129.57, 123.32, 122.61, 121.43, 120.09, 119.86. ESI-MS: M/Z [M+H]^+^ 327.1365.

(**6b**) 3-chloro-4-[(5-chloro-2-methoxybenzoyl)amino]benzoic acid

Compound **6b** was obtained using general procedure B starting from **5b** (820 mg, 2.3 mmol) in 96% (779 mg) yield as a white powder which was used without further purification.

(**6c**) 3-chloro-4-{[2-(benzyloxy)-5-chlorobenzoyl]amino}- benzoic acid

Compound **6c** was obtained using general procedure B starting from **5c** (107 mg, 0.25 mmol) in 96% (100 mg) yield as a white powder which was used without further purification.

(**7**) 5-chloro-*N*-[2-chloro-4-(methylcarbamoyl)phenyl]-2-hydroxybenzamide

Compound **7** was obtained using general procedure C starting from **6b** (200 mg, 0.6 mmol) and methylamine HCl, followed by general procedure D resulting in 119 mg (0.35 mmol) of white powder (58% over 2 steps). ^1^H NMR (500 MHz, DMSO-*d*_6_) δ 12.33 (s, 1H), 11.05 (s, 1H), 8.54 (dd, *J* = 22.9, 7.1 Hz, 2H), 8.05–7.96 (m, 2H), 7.86 (d, *J* = 8.7 Hz, 1H), 7.52 (dd, *J* = 8.7, 2.5 Hz, 1H), 7.09 (d, *J* = 8.8 Hz, 1H), 2.79 (d, *J* = 4.2 Hz, 3H). ^13^C NMR (126 MHz, DMSO-*d*_6_) δ 165.19, 162.96, 155.68, 137.82, 134.11, 131.29, 130.40, 128.56, 127.31, 124.11, 123.10, 121.85, 120.16, 119.59, 26.75. ESI-MS: M/Z [M+H]^+^ 339.0287.

(**8**) methyl [3-chloro-4-(5-chloro-2-hydroxybenzamido)benzamido]acetate

Compound **8** was obtained using general procedure C starting from **6b** (200 mg, 0.6 mmol) and glycine methyl ester HCl, followed by general procedure D resulting in 132 mg (0.33 mmol) of white powder (55% over 2 steps). ^1^H NMR (500 MHz, DMSO-*d*_6_) δ 12.36 (s, 1H), 11.10 (s, 1H), 9.06 (t, *J* = 5.8 Hz, 1H), 8.61 (d, *J* = 8.6 Hz, 1H), 8.03 (m, 2H), 7.92 (dd, *J* = 8.6, 2.1 Hz, 1H), 7.53 (dd, *J* = 8.7, 2.8 Hz, 1H), 7.10 (d, *J* = 8.7 Hz, 1H), 4.03 (s, 2H), 3.67 (s, 3H). ^13^C NMR (126 MHz, DMSO-*d*_6_) δ 170.74, 165.25, 162.97, 155.67, 138.30, 134.14, 130.42, 130.26, 128.78, 127.61, 124.13, 123.09, 121.82, 120.15, 119.59, 52.25, 41.72. ESI-MS: M/Z [M+H]^+^ 397.0324.

(**9**) methyl (4*S*)-5-amino-4-[3-chloro-4-(5-chloro-2-hydroxybenzamido)benzamido]-5-oxopentanoate

Compound **9** was obtained using general procedure C starting from **6b** (200 mg, 0.6 mmol) and L-Glutamic Acid γ-Methyl Ester α-Amide Hydrochloride followed by general procedure D resulting in 146 mg (0.31 mmol) of white powder (52% over 2 steps). ^1^H NMR (500 MHz, DMSO-*d*_6_) δ. 12.42 (s, 1H), 11.09 (s, 1H), 8.51 (m, 2H), 8.09 (s, 1H), 7.92 (m, 2H), 7.42 (m, 2H), 6.09 (m, 2H) 4.32 (m, 1H), 3.52 (s, 1H), 2.23 (m, 2H), 1.98 (m, 1H), 1.78 (m, 1H). ^13^C NMR (126 MHz, DMSO-*d*_6_) δ 173.61, 173.32, 165.05, 163.04, 155.90, 138.07, 134.11, 130.78, 130.39, 129.03, 124.94, 123.98, 122.90, 121.66, 120.15, 119.65, 53.18, 51.80, 30.77, 27.28. ESI-MS: M/Z [M+H]^+^ 468.0712.

(**10**) (4*S*)-5-amino-4-[3-chloro-4-(5-chloro-2-hydroxybenzamido)benzamido]-5-oxopentanoic acid

Compound **10** was obtained using general procedure B starting from **9** (100 mg, 0.21 mmol) resulting in 78 mg (0.17 mmol) of off-white powder (81%). ^1^H NMR (500 MHz, Methanol-*d*_4_) δ 8.65 (d, *J* = 8.6 Hz, 1H), 8.05 (d, *J* = 2.7 Hz, 1H), 8.02 (d, *J* = 2.0 Hz, 1H), 7.86 (dd, *J* = 8.7, 2.0 Hz, 1H), 7.41 (dd, *J* = 8.7, 2.8 Hz, 1H), 6.98 (d, *J* = 8.7 Hz, 1H), 4.58 (dd, *J* = 9.2, 5.0 Hz, 1H), 2.48 (t, *J* = 7.4 Hz, 2H), 2.26–2.20 (m, 1H), 2.12–2.05 (m, 1H). ^13^C NMR (126 MHz, Methanol-*d*_4_) δ 175.29, 175.07, 166.73, 163.32, 155.04, 138.27, 133.35, 130.22, 129.86, 128.44, 126.60, 124.87, 123.16, 121.17, 119.70, 118.10, 53.27, 30.06, 26.79. ESI-MS: M/Z [M+H]^+^ 454.0515.

(**11**) 5-chloro-*N*-(4-{[(2S)-1,4-diamino-1-oxobutan-2-yl]carbamoyl}phenyl)-2-hydroxybenzamide

Compound **11** was obtained using general procedure C starting from **6c** (100 mg, 0.3 mmol) and L-diaminobutyric acid γ- Carbobenzoxy-α-Amide hydrochloride followed by general procedure E resulting in 43 mg (0.10 mmol) of brown powder (34% over 2 steps). 1H NMR (500 MHz, DMSO-*d*_6_) δ 12.52–12.43 (m, 1H), 11.12 (s, 1H), 8.71 (d, J = 7.8 Hz, 1H), 8.60 (d, *J* = 8.4 Hz, 1H), 8.14 (s, 1H), 7.99 (d, *J* = 2.3 Hz, 1H), 7.95 (d, *J* = 8.7 Hz, 1H), 7.77–7.69 (m, 3H), 7.57–7.50 (m, 2H,), 7.23 (s, 1H, 12-b), 7.12 (d, *J* = 8.6 Hz, 1H), 4.51–4.46 (m, 1H), 2.92–2.85 (m, 2H), 2.11 (dd, *J* = 14.1, 6.8 Hz, 1H), 1.96 (dd, *J* = 14.6, 7.3 Hz, 1H). 13C NMR (126 MHz, DMSO-*d*_6_) δ 173.09, 165.18, 163.00, 155.72, 138.15, 134.17, 130.62, 130.38, 129.09, 128.02, 124.09, 122.87, 121.64, 120.12, 119.62, 51.44, 36.97, 29.97 ESI-MS: M/Z [M+H]^+^ 425.0761.

(**12**) (2*S*)-2-[3-chloro-4-(5-chloro-2-hydroxybenzamido)benzamido]pentanedioic acid

Compound **12** was obtained using general procedure C starting from **6c** (100 mg, 0.3 mmol) and Di-tert-butyl (S)-2-Aminopentanedioate hydrochloride followed by general procedure E and then general procedure B resulting in 22 mg (0.05 mmol) of white powder (15% over 3 steps). ^1^H NMR (500 MHz, Methanol-*d*_4_) δ 8.73 (d, *J* = 8.8 Hz, 1H), 8.02 (d, *J* = 2.1 Hz, 1H), 7.86–7.79 (m, 2H), 7.08 (dd, *J* = 8.8, 3.1 Hz, 1H), 6.67 (d, *J* = 8.9 Hz, 1H), 4.39 (dd, *J* = 8.3, 4.4 Hz, 1H), 2.35–2.21 (m, 3H), 2.13–2.08 (m, 1H). ^13^C NMR (126 MHz, Methanol-*d*_4_) δ 180.97, 177.83, 168.80, 167.48, 166.23, 139.73, 132.64, 129.31, 128.45, 128.32, 128.25, 125.99, 123.53, 120.97, 119.21, 116.99, 56.06, 34.39, 29.03. ESI-MS: M/Z [M+Na]^+^ 477.0291.

(**13**) 5-chloro-*N*-{2-chloro-4-[(prop-2-yn-1-yl)carbamoyl]phenyl}-2-hydroxybenzamide

Compound **13** was obtained using general procedure C starting from **6b** 500 mg, 1.5 mmol) and propargylamine followed by general procedure D resulting in 267 mg (0.74 mmol) of white powder (49% over 2 steps) which was used without further purification.

(**14**) methyl {4-[({3-chloro-4-[(5-chloro-2-hydroxybenzoyl)amino]benzoyl}amino)ethyl]-1*H*-1,2,3-triazol-1-yl}acetate

Compound **14** was obtained using general procedure F starting from **13** (55 mg, 0.15 mmol) and ethyl 2-azidoacetate resulting in 23 mg (0.05 mmol) of off-white powder (33%).^1^H NMR (300 MHz, DMSO-*d*_6_) δ 12.35 (s, 1H), 11.08 (s, 1H), 9.16 (t, *J* = 5.8 Hz, 1H), 8.58 (d, *J* = 8.7 Hz, 1H), 8.08 (d, *J* = 2.0 Hz, 1H), 8.00–7.88 (m, 3H), 7.53 (dd, *J* = 8.7, 2.8 Hz, 1H), 7.10 (d, *J* = 8.8 Hz, 1H), 5.35 (s, 2H), 4.54 (d, *J* = 5.7 Hz, 2H), 4.17 (q, *J* = 7.1 Hz, 2H), 1.22–1.18 (m, 3H). ^13^C NMR (126 MHz, DMSO-*d*_6_) δ 167.72, 164.75, 162.95, 155.66, 145.45, 138.06, 134.13, 130.81, 130.40, 128.77, 127.59, 124.93, 124.11, 123.04, 121.76, 120.14, 119.58, 61.88, 50.74, 35.33, 14.44. ESI-MS: M/Z [M+H]^+^ 492.0779.

(**15**) {4-[({3-chloro-4-[(5-chloro-2-hydroxybenzoyl)amino]benzoyl}amino)methyl]-1*H*-1,2,3-triazol-1-yl}acetic acid

Compound **15** was obtained using general procedure B starting from **13** (15 mg, 0.03 mmol) resulting in 12 mg (0.025 mmol) of off-white powder (86%).^1^H NMR (500 MHz, DMSO-*d*_6_) δ 11.29 (s, 1H), 9.17 (t, *J* = 5.8 Hz, 1H), 8.59 (d, *J* = 8.7 Hz, 1H), 8.08 (d, *J* = 2.0 Hz, 1H), 7.97 (d, *J* = 3.1 Hz, 2H), 7.92 (dd, *J* = 8.6, 2.0 Hz, 1H), 7.50 (dd, *J* = 8.7, 2.9 Hz, 1H), 7.11 (dd, *J* = 9.2, 5.0 Hz, 1H), 5.21 (s, 2H), 4.53 (d, *J* = 5.7 Hz, 2H). ^13^C NMR (126 MHz, DMSO-*d*_6_) δ 164.81, 163.13, 156.33, 145.34, 138.19, 134.03, 130.68, 130.31, 128.77, 127.57, 124.86, 123.63, 122.97, 121.68, 119.79, 53.08, 35.33. ESI-MS: M/Z [M+H]^+^ 464.0523.

(**16**) {4-[({3-chloro-4-[(5-chloro-2-hydroxybenzoyl)amino]benzoyl}amino)methyl]-1*H*-1,2,3-triazol-1-yl}benzoic acid

Compound **16** was obtained using general procedure F starting from **13** (55 mg, 0.15 mmol) and 4-azido benzoic acid resulting in 42 mg (0.08 mmol) of off-white powder (53%). ^1^H NMR (500 MHz, DMSO-*d*_6_) δ 13.20 (s, 1H), 12.52 (s, 1H), 11.12 (s, 1H), 9.23 (s, 1H), 8.82 (d, *J* = 7.1 Hz, 1H), 8.59 (d, *J* = 8.5 Hz, 1H), 8.16–8.06 (m, 5H), 8.03–7.93 (m, 2H), 7.52 (d, *J* = 8.4 Hz, 1H), 7.19 (d, *J* = 8.4 Hz, 1H), 4.63 (d, *J* = 5.5 Hz, 2H). ^13^C NMR (126 MHz, DMSO-*d*_6_) δ 166.83, 164.96, 164.87, 138.09, 134.09, 131.55, 131.52, 130.94, 130.35, 128.87, 127.72, 124.00, 122.96, 121.85, 121.69, 121.19, 120.13, 35.38. ESI-MS: M/Z [M+H]^+^ 526.0617.

(**17**) 3-{4-[({3-chloro-4-[(5-chloro-2-hydroxybenzoyl)amino]benzoyl}amino)methyl]-1*H*-1,2,3-triazol-1-yl-phenyl}propionic acid

Compound **17** was obtained using general procedure F starting from **13** (55 mg, 0.15 mmol) and 3-(4-Azidophenyl)propanoic acid resulting in 51 mg (0.09 mmol) of off-white powder (61%). ^1^H NMR (500 MHz, DMSO-*d*_6_) δ 12.36 (s, 1H), 12.17 (s, 1H), 11.09 (s, 1H), 9.17 (t, *J* = 5.6 Hz, 1H), 8.66 (s, 1H), 8.59 (d, *J* = 8.7 Hz, 1H), 8.11 (d, *J* = 1.9 Hz, 1H), 8.02–7.93 (m, 2H), 7.81 (d, *J* = 8.1 Hz, 2H), 7.53 (dd, *J* = 8.9, 2.8 Hz, 1H), 7.44 (d, *J* = 8.1 Hz, 2H), 7.10 (d, *J* = 8.7 Hz, 1H), 4.62 (d, *J* = 5.4 Hz, 2H), 2.90 (t, *J* = 7.6 Hz, 2H), 2.60 (t, *J* = 7.6 Hz, 2H). ^13^C NMR (126 MHz, DMSO-*d*_6_) δ 174.07, 164.81, 162.96, 155.67, 146.37, 141.97, 138.04, 135.33, 134.12, 130.86, 130.39, 130.07, 130.07, 128.85, 127.69, 124.10, 123.02, 121.74, 121.61, 120.38, 120.37, 120.14, 119.58, 35.40, 35.36, 30.24. ESI-MS: M/Z [M+H]^+^ 554.0965.

(**18**) *N*-(2-chloro-4-nitrophenyl)-2,4-dihydroxybenzamide

Compound **18** was obtained using general procedure A starting from 2,4-Dimethoxybenzoic acid (182mg, 1 mmol) and 2-chloro-4-nitro aniline (172 mg, 1 mmol) followed by general procedure D, resulting in 120 mg (0.39 mmol) pale orange powder (39% over 2 steps)^1^H NMR (500 MHz, DMSO-*d*_6_) δ 11.99 (s, 1H), 11.21 (s, 1H), 10.31 (s, 1H), 8.86 (d, *J* = 9.2 Hz, 1H), 8.42 (d, *J* = 2.6 Hz, 1H), 8.28 (dd, *J* = 9.3, 2.6 Hz, 1H), 7.89 (d, *J* = 8.6 Hz, 1H), 6.49–6.43 (m, 2H). ^13^C NMR (126 MHz, DMSO-*d*_6_) δ 164.27, 163.42, 158.44, 142.44, 142.38, 133.45, 125.23, 124.35, 122.32, 120.77, 110.04, 109.17, 103.10. ESI-MS (negative mode): M/Z [M−H]^−^ 307.0388.

(**19**) *N*-(2-chloro-4-nitrophenyl)-2,4,5-trihydroxybenzamide

Compound **19** was obtained using general procedure A starting from 2,4,5-Trimethoxybenzoic acid (212 mg, 1 mmol) and 2-chloro-4-nitro aniline (172 mg, 1 mmol) followed by general procedure D, resulting in 175 mg (0.54 mmol) pale yellow powder (54% over 2 steps) ^1^H NMR (500 MHz, DMSO-*d*_6_) δ 11.26 (s, 2H), 9.88 (s, 1H), 8.83–8.80 (m, 2H), 8.35 (d, *J* = 2.6 Hz, 1H), 8.21 (dd, *J* = 9.3, 2.7 Hz, 1H), 7.36 (s, 1H), 6.45 (s, 1H). ^13^C NMR (126 MHz, DMSO-*d*_6_) δ 164.25, 152.17, 150.86, 142.52, 142.24, 139.69, 125.21, 124.33, 122.04, 120.49, 116.63, 108.86, 104.09 ESI-MS (negative mode): M/Z [M−H]^−^ 323.0201.

(**20**) 3-chloro-4-[(5-chloro-2-hydroxybenzoyl)amino]-*N*-(3,4-dihydroxybenzyl)benzamide

Compound **20** was obtained using general procedure C starting from **6b** (100 mg, 0.3 mmol) and 1-(3,4-dimethoxyphenyl)methanamine followed by general procedure D resulting in 52 mg (0.12 mmol) of white powder (39% over 2 steps).^1^H NMR (500 MHz, DMSO-*d*_6_) δ 12.37 (s, 1H), 11.11 (s, 1H), 8.98 (t, *J* = 6.0 Hz, 1H), 8.84 (s, 1H), 8.71 (s, 1H), 8.58 (d, *J* = 8.7 Hz, 1H), 8.14–8.06 (m, 1H), 7.99 (d, *J* = 2.9 Hz, 1H), 7.93 (d, *J* = 8.8 Hz, 1H), 7.53 (dd, *J* = 8.8, 2.9 Hz, 1H), 7.10 (d, *J* = 8.7 Hz, 1H), 6.73 (s, 1H), 6.67 (d, *J* = 8.0 Hz, 1H), 6.58 (d, *J* = 8.0 Hz, 1H), 4.31 (d, *J* = 5.8 Hz, 2H). ^13^C NMR (126 MHz, DMSO-*d*_6_) δ 164.50, 162.98, 155.77, 145.54, 144.64, 137.93, 134.11, 131.20, 130.70, 130.38, 128.71, 127.54, 124.03, 123.06, 121.80, 120.14, 119.61, 118.76, 115.77, 115.38, 42.83. ESI-MS: M/Z [M+H]^+^ 447.0510.

(**21**) 3-chloro-4-[(5-chloro-2-hydroxybenzoyl)amino]-*N*-[2-(3,4-dihydroxyphenyl)ethyl]benzamide

Compound **21** was obtained using general procedure C starting from **6b** (100 mg, 0.3 mmol) and 2-(3,4-dimethoxyphenyl)ethan-1-amine followed by general procedure D resulting in 67 mg (0.15 mmol) of white powder (50% over 2 steps).^1^H NMR (500 MHz, DMSO-*d*_6_) δ 12.37 (s, 1H), 11.09 (s, 1H), 8.76 (s, 1H), 8.70–8.53 (m, 3H), 8.00 (d, *J* = 14.3 Hz, 2H), 7.87 (d, *J* = 8.7 Hz, 1H), 7.52 (d, *J* = 8.7 Hz, 1H), 7.10 (d, *J* = 8.8 Hz, 1H), 6.70–6.58 (m, 2H), 6.48 (d, *J* = 8.0 Hz, 1H), 3.41 (s, 2H), 2.66 (t, *J* = 7.5 Hz, 2H). ^13^C NMR (126 MHz, DMSO-*d*_6_) δ 164.62, 162.96, 155.76, 145.53, 144.00, 137.84, 134.08, 131.37, 130.66, 130.38, 128.61, 127.39, 124.04, 123.04, 121.78, 120.14, 119.70, 119.59, 116.46, 115.96, 41.85, 35.03. ESI-MS: M/Z [M+H]^+^ 461.0597.

NMR spectra can be found in supplementary materials. 

### 3.3. Antibacterial Activity

#### 3.3.1. Antimicrobial Susceptibility Assay

Bacterial samples for this research were sourced from the American Type Culture Collection (ATCC), the Canadian National Intensive Care Unit (CAN-ICU) surveillance study [22], and the Canadian Ward (CANWARD) surveillance study [23]. Clinical samples from the CAN-ICU and CANWARD studies were taken from patients with suspected infectious diseases in participating medical centers across Canada during the study period. The antibacterial properties of the compounds were evaluated using the microbroth dilution technique as per the Clinical and Laboratory Standards Institute (CLSI) guidelines [24]. Bacterial cultures were cultured overnight and then diluted in saline to a 0.5 McFarland standard. This was further diluted 1:50 in Cation-adjusted Mueller-Hinton broth (CAMHB) to yield a concentration of about 5 × 10^5^ CFU/mL. Tests were conducted in 96-well plates. The agents were serially diluted in CAMHB and incubated with bacterial samples at 37 °C for 18 h. The minimum inhibitory concentration (MIC) was determined as the lowest concentration preventing visible bacterial growth, confirmed using an EMax Plus microplate reader (Molecular Devices, San Jose, CA, USA) at 590 nm. CAMHB, with or without bacterial cells, served as positive and negative controls, respectively.

#### 3.3.2. Checkerboard Assay

The experiment was conducted in 96-well plates as described previously [25]. One agent underwent a 2-fold serial dilution along the *x*-axis, while the other was similarly diluted along the *y*-axis, resulting in a matrix where each well held both agents at varying concentrations. Bacterial cultures, cultivated overnight, were diluted in saline to achieve a 0.5 McFarland turbidity. This was then further diluted 1:50 in CAMHB, and each well was inoculated, reaching an approximate concentration of 5 × 10^5^ CFU/mL. Wells containing only CAMHB, with or without bacteria, served as the positive and negative controls, respectively. The plates were incubated at 37 °C for 18 h and checked for visible turbidity, verified with an EMax Plus microplate reader (Molecular Devices, USA) at 590 nm. The fractional inhibitory concentration (FIC) for each agent was determined by dividing the MIC of the compound with colistin by its MIC when alone. Similarly, the FIC for colistin was determined by dividing its MIC with each compound by its standalone MIC. The FIC index was derived by adding both FIC values together. FIC indices were classified as synergistic (≤0.5), indifferent (0.5 < x ≤ 4), or antagonistic (>4).

### 3.4. Cell Proliferation Assay

Cell proliferation was assessed by quantifying changes in cell numbers using the CyQuant cell proliferation assay (Invitrogen, Waltham, MA, USA), essentially as described by the manufacturer. OVCAR-3 and COV362 cells were seeded in 96-well plates (7500 cells/well) in a volume of 100 µL. Wells with only media and no cells served as blanks. The plates were maintained in a 5% CO_2_ incubator at 37 °C. Two additional plates with cells and blank wells were prepared as control plates (did not receive the drug treatment) for determination of the cell numbers on the day of the addition of the drugs (day 0) and after the 48 h incubation absolute cell numbers from representative wells from these plates were determined with a Coulter ZM counter, while the remainder wells were processed for the CyQuant assay as described below. When cells were in the log phase, drug solutions were added to the test plates to yield final concentrations of 0–100 µM. The control plates received the media with the vehicle. After 48 h incubation, the media was removed, and the plates were placed at −80 °C for 7 days. The plates were allowed to thaw to room temperature, and CyQuant reagent in lysis buffer (200 µL) was added to each well. Fluorescence was measured on a SpectraMax M2 microplate reader (Molecular Devices, USA) at excitation and emission wavelengths of 480 and 520 nm, respectively. The results were expressed as percent cell number relative to initial cell number for each concentration.

## 4. Conclusions

A series of niclosamide analogs were synthesized to perform an SAR with an overall goal of replacing the nitro group of niclosamide to reduce toxicity while maintaining the ability of niclosamide to synergize with colistin against GNB. A small library of compounds was produced and their ability to synergize with colistin was observed. It was found that the nitro group on niclosamide can be replaced by an amine, a methyl ester, or an azide while still retaining colistin-potentiating activity. We also showed that the phenol group of niclosamide was necessary for synergy with colistin, but that the addition of multiple phenols led to a complete loss of synergy. The methyl ester analog (**5a**) was assessed against a panel of MDR clinical isolates and was shown to reduce colistin MIC to below CLSI breakpoint values, even against highly resistant isolates. The amine and methyl ester compounds were also found to be less toxic to eukaryotic cells. One significant challenge that needs to be overcome is the likely metabolic instability of the ester in compound **5a**, as esters are known to be prone to hydrolysis in vivo [26], especially given that the carboxylic acid derivative **6** displayed no synergy with colistin. Nonetheless, this work provides important insights into synthetic strategies for the future development of new niclosamide derivatives, and that modification to the nitro group of niclosamide may be a viable strategy of reducing toxicity. 

## Figures and Tables

**Table 1:** Colistin MIC in combination with 4 μM of compounds **1**–**3** against four colistin-resistant strains of GNB. KP = *Klebsiella pneumoniae,* EC = *Escherichia coli*.

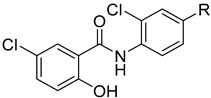					
Colistin MIC with 4 μM Compound (μg/mL)					
Compound	R	KP113250	KP113254	EC94393	EC94474
Niclosamide	NO_2_	0.25	0.5	0.25	0.5
**1**	NH_2_	16	32	0.5	2
**2**	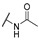	256	256	8	16
**3**	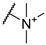	256	256	8	16
Colistin MIC alone		256	256	8	16

**Table 2:** Colistin MIC in combination with 4 μM of compounds **4**–**12** and **14**–**17** against four colistin-resistant strains of GNB. KP = *Klebsiella pneumoniae,* EC = *Escherichia coli*.

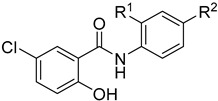						
Colistin MIC with 4 μM Compound (μg/mL)						
Compound	R^1^	R^2^	KP113250	KP113254	EC94393	EC94474
Niclosamide	Cl	NO_2_	0.25	0.5	0.25	0.5
**4**	H	N_3_	0.25	0.5	0.016	0.25
**5a**	Cl	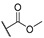	1	1	0.25	1
**6**	Cl	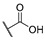	256	256	8	16
**7**	Cl	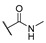	256	256	8	16
**8**	Cl	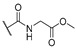	256	256	8	16
**9**	Cl	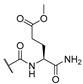	256	256	8	16
**10**	Cl	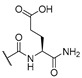	256	256	8	16
**11**	Cl	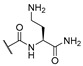	256	256	8	16
**12**	Cl	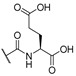	256	256	8	16
**14**	Cl	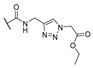	256	256	8	16
**15**	Cl	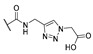	256	256	8	16
**16**	Cl	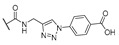	256	256	8	16
**17**	Cl	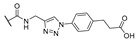	256	256	8	16
Colistin MIC alone			256	256	8	16

**Table 3:** Colistin MIC in combination with 4 μM of polyphenol compounds **18**–**21** against four colistin-resistant strains of GNB. KP = *Klebsiella pneumoniae,* EC = *Escherichia coli*.

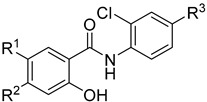							
Colistin MIC with 4 μM Compound (μg/mL)							
Compound	R^1^	R^2^	R^3^	KP113250	KP113254	EC94393	EC94474
Niclosamide	Cl	H	NO_2_	0.25	0.5	0.25	0.5
**18**	H	OH	NO_2_	256	256	8	16
**19**	OH	OH	NO_2_	256	256	8	16
**20**	Cl	H	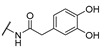	256	256	8	16
**21**	Cl	H	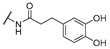	256	256	8	16
Colistin MIC alone				256	256	8	16

**Table 4:** Synergy between compound **5a** and colistin against a panel of wild-type and clinical isolates of *P. aeruginosa*.

Strain	MIC_Col_	MIC_Combi_	MIC_5a_	MIC_Combi_	FICI	Interpretation
PAO1	1	0.25	>256	0.25	0.250 < x < 0.251	Synergy
PA095	0.25	0.016	>256	16	0.063 < x < 0.125	Synergy
PA259	0.25	0.25	>256	1	1.000 < x < 1.004	Additive
PA260	0.25	0.125	>256	1	0.500 < x < 0.504	Additive
PA264	1	0.25	>256	0.5	0.250 < x < 0.252	Synergy
PA262	4	0.25	>256	1	0.063 < x < 0.066	Synergy
PA100036	2	0.25	>256	1	0.125 < x < 0.129	Synergy
PA101885	4	0.5	>256	1	0.125 < x < 0.129	Synergy
PA91433	4	0.5	>256	0.25	0.125 < x < 0.126	Synergy
PA101243	1024	0.5	>256	1	0.0005 < x < 0.0045	Synergy

**Table 5:** Synergy between compound **5a** and colistin against a panel of wild-type and clinical isolates of *A. baumannii*.

Strain	MIC_Col_	MIC_Combi_	MIC_5a_	MIC_Combi_	FICI	Interpretation
*A. baumannii* ATCC 17978	1	0.25	>256	0.25	0.250 < x < 0.251	Synergy
*A baumannii* 110193	4	0.5	>256	0.25	0.125 < x < 0.126	Synergy
AB027	1024	0.5	>256	1	0.0005 < x < 0.004	Synergy
AB031	0.25	0.015625	>256	16	0.063 < x < 0.125	Synergy
LAC-4	0.25	0.25	>256	1	1.000 < x < 1.004	Additive

## Data Availability

Data are contained within this manuscript and Supplementary Materials.

## Supplementary Materials

The following supporting information can be downloaded at: https://www.mdpi.com/article/10.3390/antibiotics13010043/s1, Figure S1–S42 NMR Spectra; Table S1 Minimum inhibitory concentration (MIC) in μg/mL of compounds **1**–**21** against 4 colistin-resistant strains of GNB. Table S2. Colistin minimum inhibitory concentration (MIC) in combination with 4 μM of compound **5b** and niclosamide against 4 colistin-resistant strains of GNB.
