# Supplementary material for: Exploring Structure–Activity Relationships of Niclosamide-Based Colistin Potentiators in Colistin-Resistant Gram-Negative Bacteria
Source: Antibiotics (Basel). 2024 Jan 3;13(1):43. doi: 10.3390/antibiotics13010043 (PMC10812775; doi:10.3390/antibiotics13010043)
Supplement: Supplementary file 1 [file antibiotics-13-00043-s001.zip › antibiotics-2796375-supplementary.pdf]

Supplementary information

Exploring Structure–Activity Relationships of Niclosamide-Based Colistin Potentiators in Colistin-Resistant Gram-Negative Bacteria

Liam Berry [1], Quinn Neale [1], Rajat Arora [1], Danyel Ramirez [1], Marc Brizuela [1], Ronald Domalaon [1], Gilbert Arthur [2] and Frank Schweizer [1,3,*]

[1] Department of Chemistry, University of Manitoba, Winnipeg, MB R3T 2N2, Canada; berryl3@myumanitoba.ca (L.B.); nealeq@myumanitoba.ca (Q.N.); arorar5@myumanitoba.ca (R.A.); ramiredm@myumanitoba.ca (D.R.); marc.brizuela@umanitoba.ca (M.B.)
[2] Department of Biochemistry and Medical Genetics, University of Manitoba, Winnipeg, MB R3E 3N4, Canada; gilbert.arthur@umanitoba.ca
[3] Department of Medical Microbiology and Infectious Diseases, University of Manitoba, Winnipeg, MB R3E 0J9, Canada
* Correspondence: frank.schweizer@umanitoba.ca

Contents

NMR spectra of compounds used for biological testing **S1**

Extended biological data **S21**

NMR Spectra of compounds used for biological testing

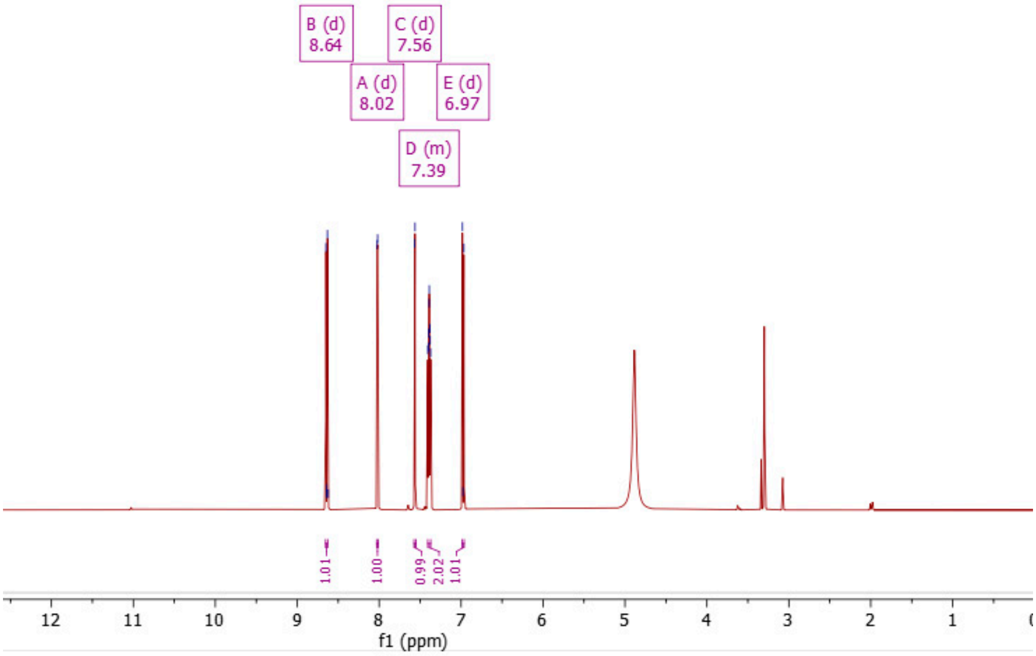

Figure S1 ^{1}H NMR spectrum of compound **1**

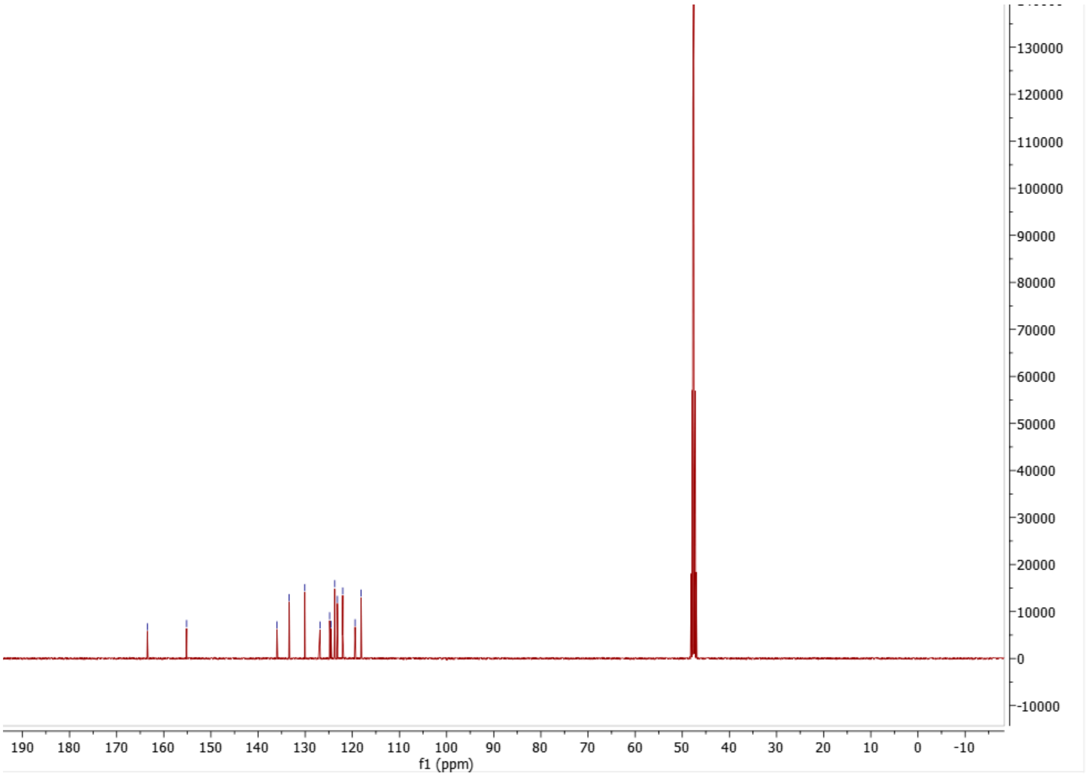

Figure S2 ^{13}C NMR spectrum of compound **1**

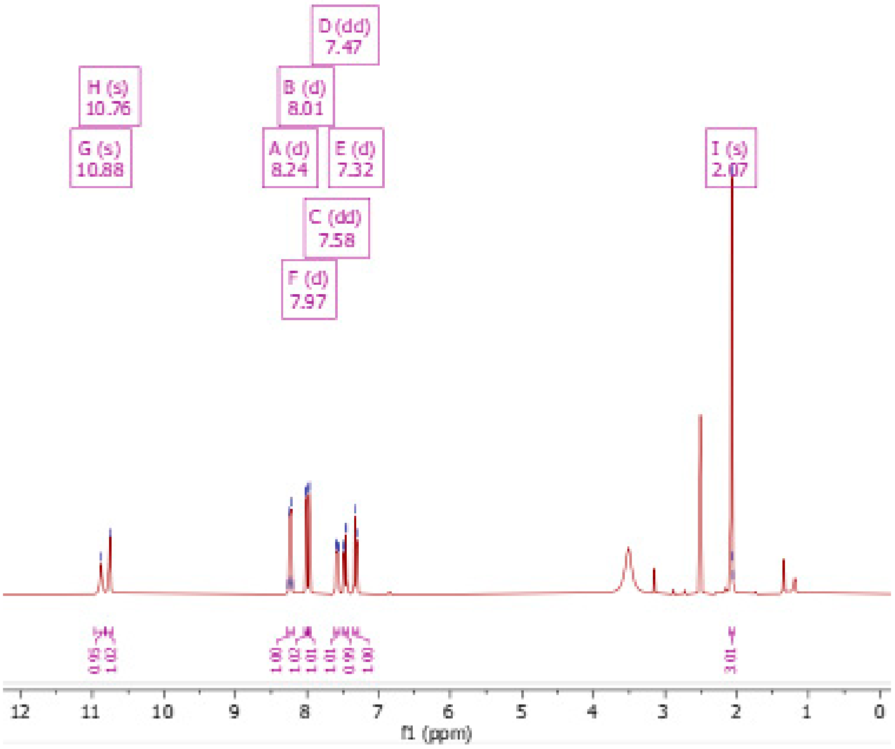

Figure S3 ^{1}H NMR spectrum of compound **2**

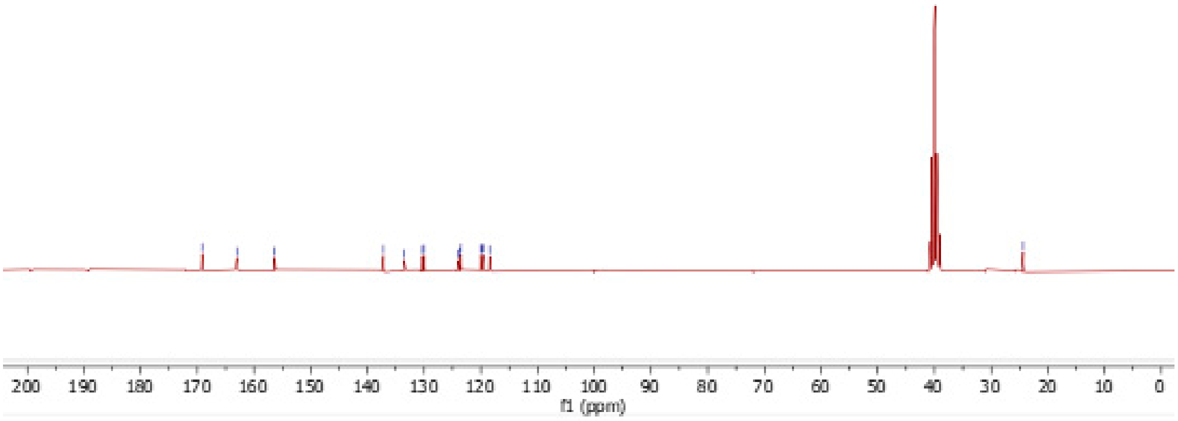

Figure S4 ^{13}C NMR spectrum of compound **2**

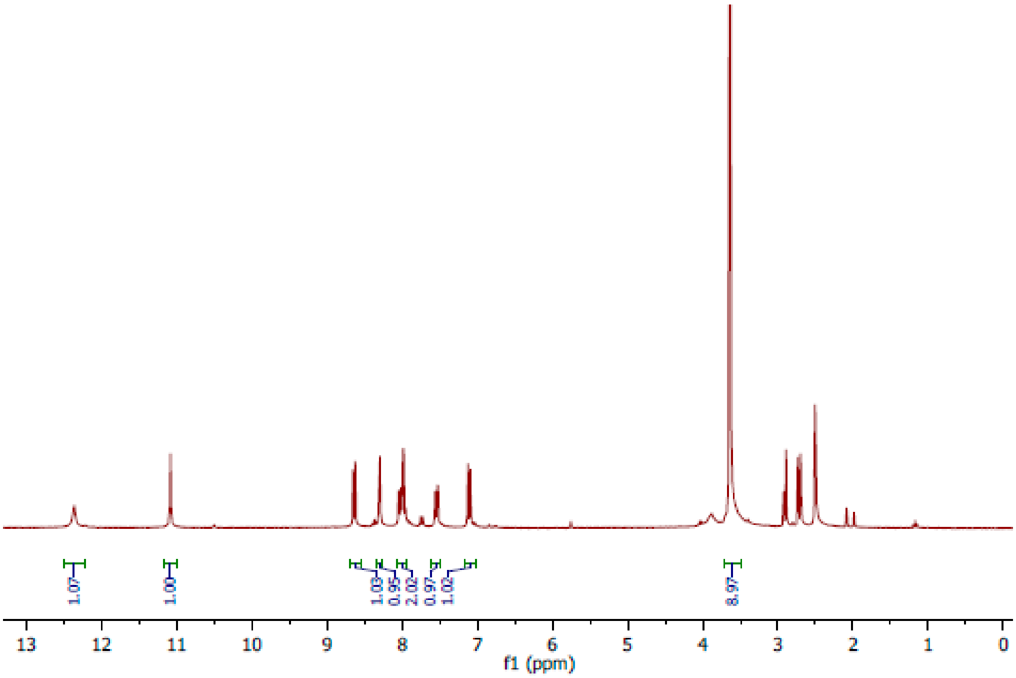

Figure S5 ^{1}H NMR spectrum of compound **3**

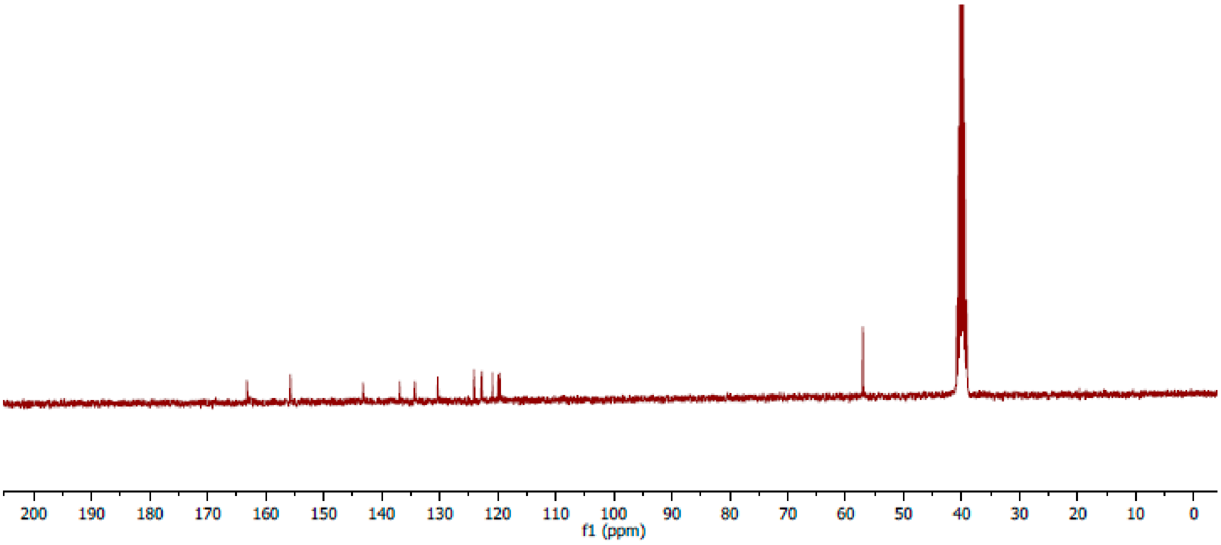

Figure S6 ^{13}C NMR spectrum of compound **3**

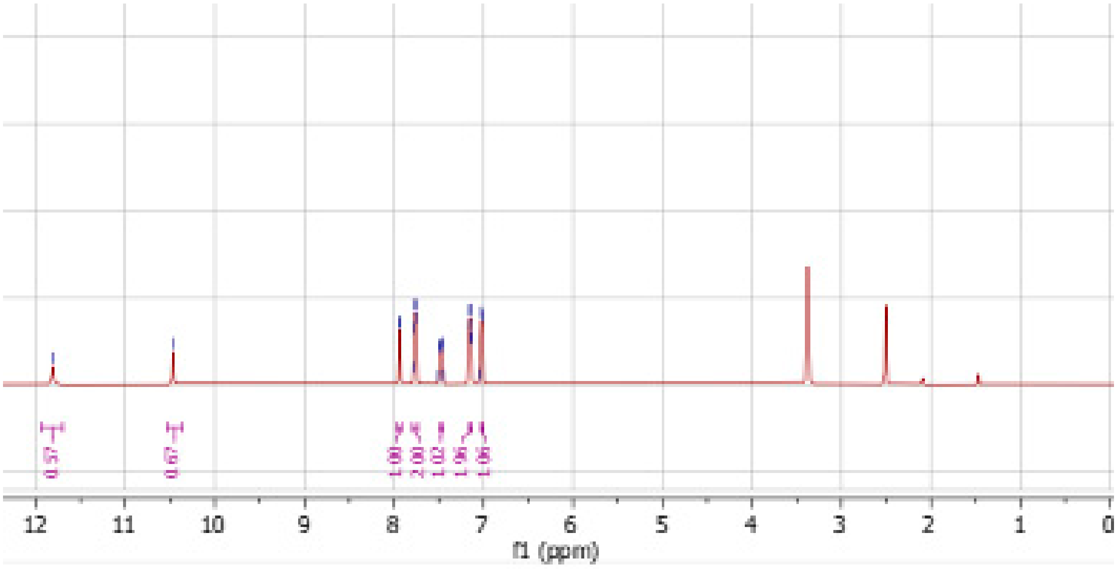

Figure S7 ^{1}H NMR spectrum of compound **4**

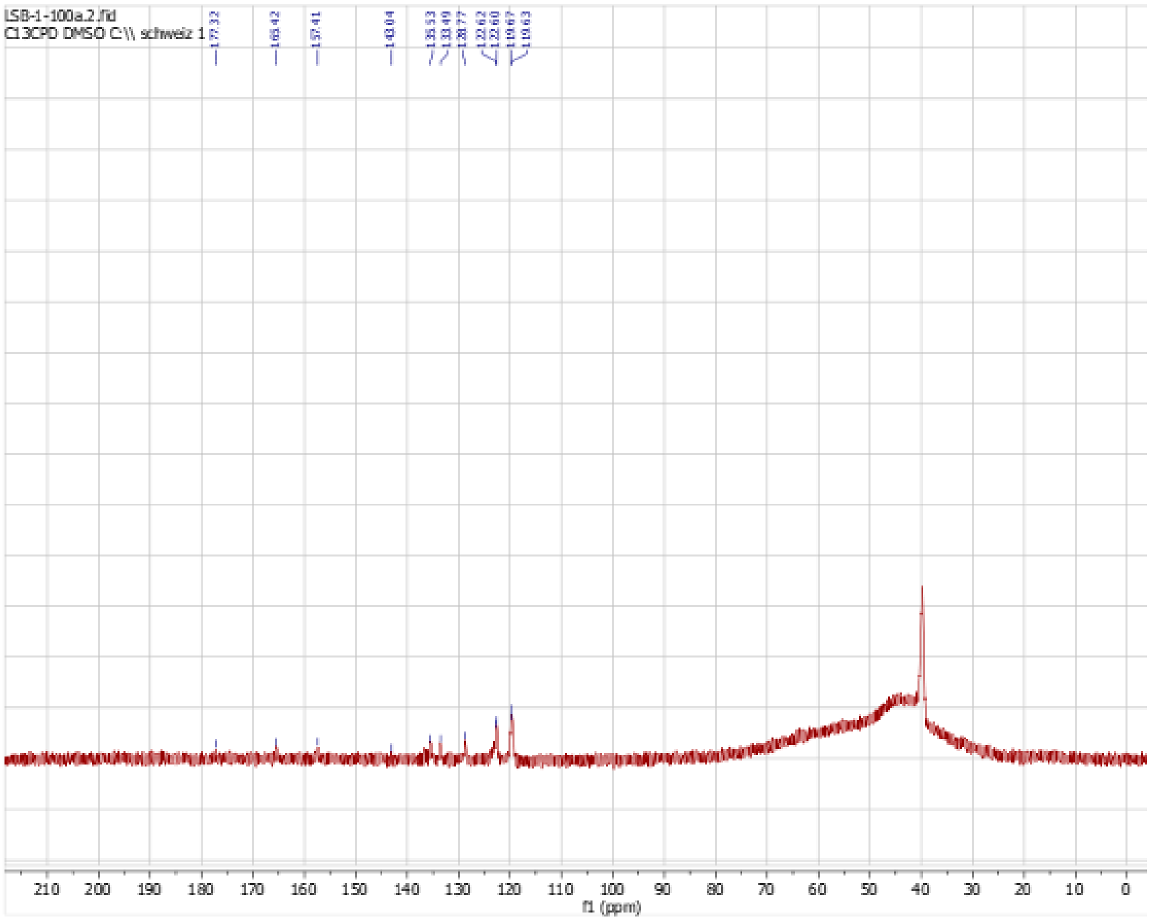

Figure S8 ^{13}C NMR spectrum of compound **4**

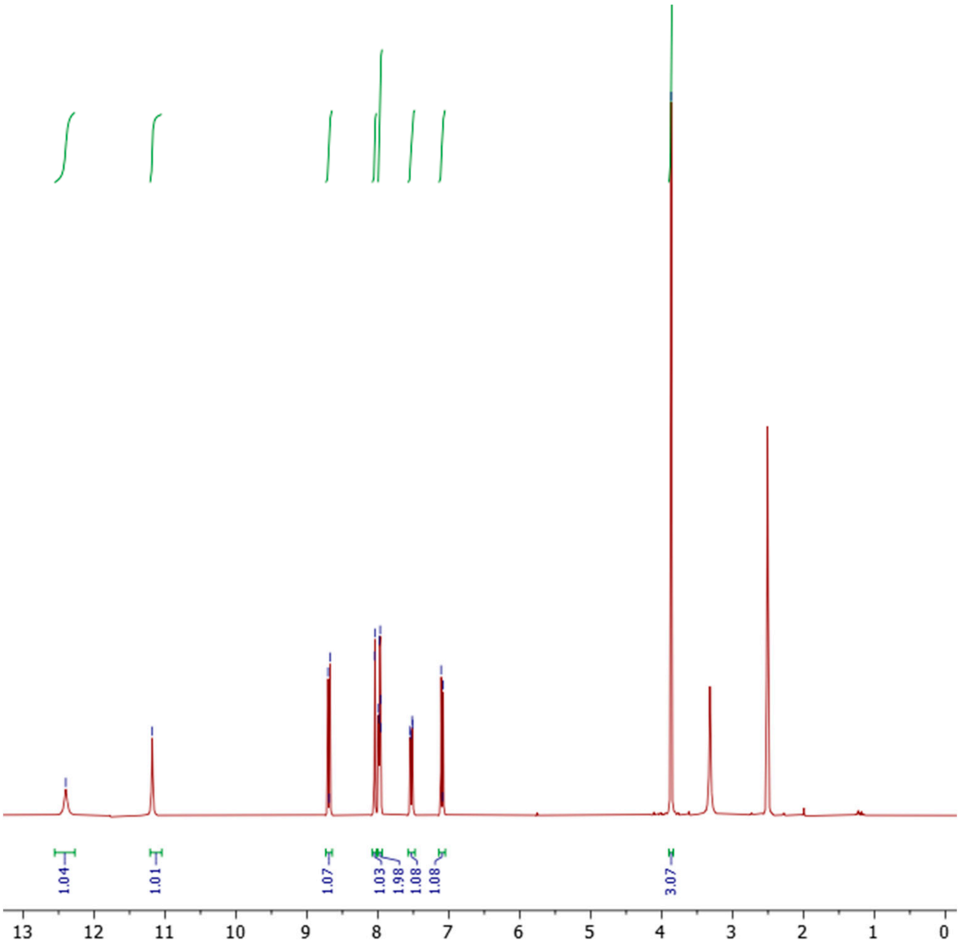

Figure S9 ^{1}H NMR spectrum of compound **5a**

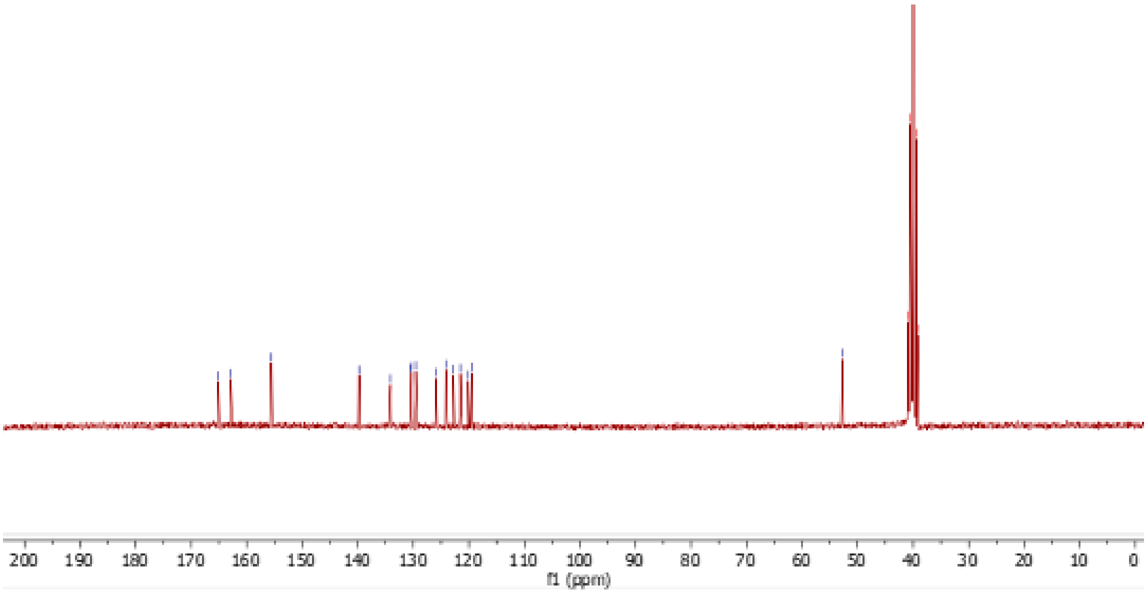

Figure S10 ^{13}C NMR spectrum of compound **5a**

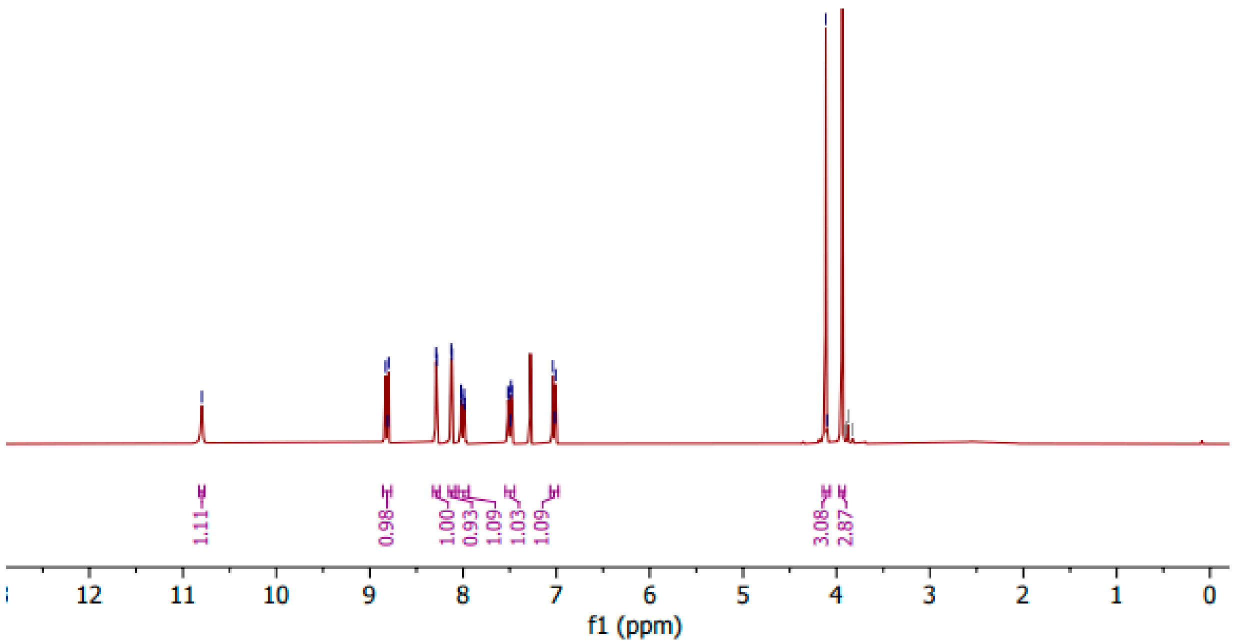

Figure S11 ^{1}H NMR spectrum of compound **5b**

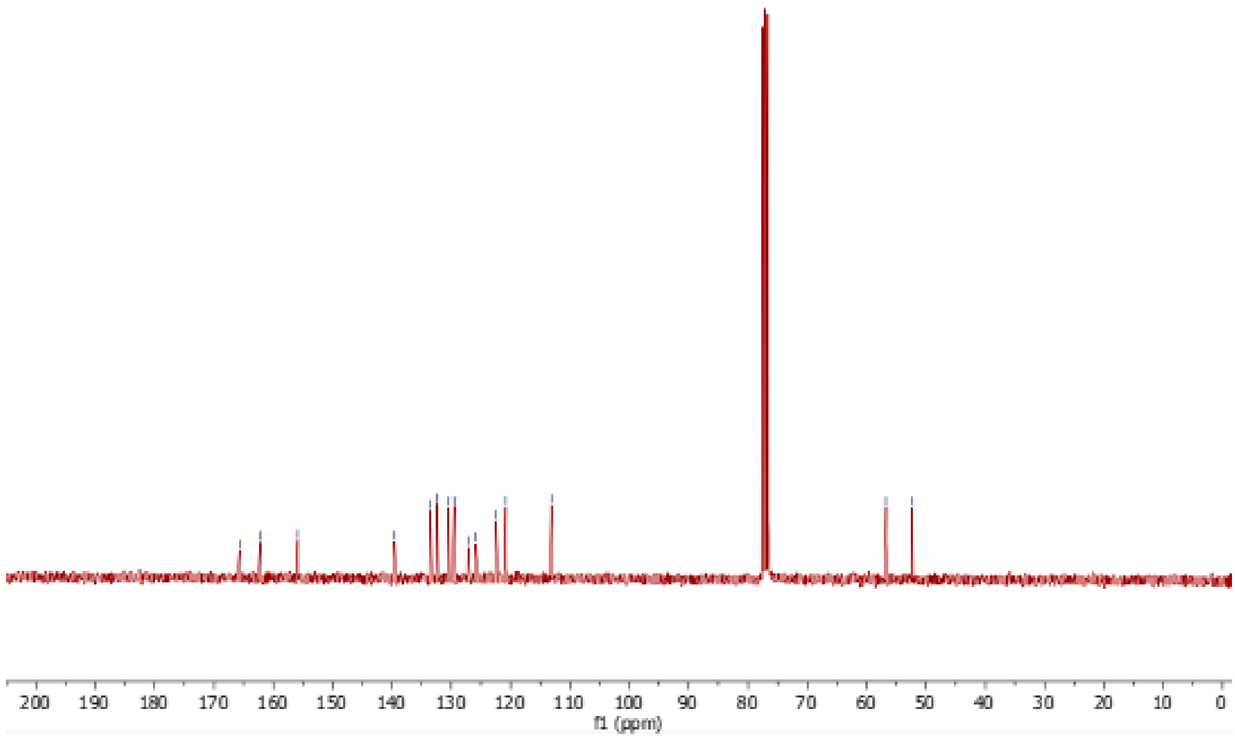

Figure S12 ^{13}C NMR spectrum of compound **5b**

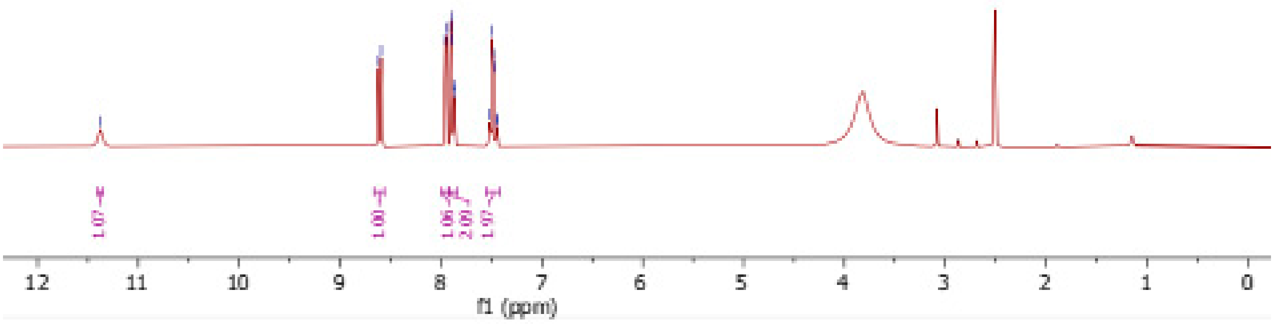

Figure S13 ^{1}H NMR spectrum of compound **6**

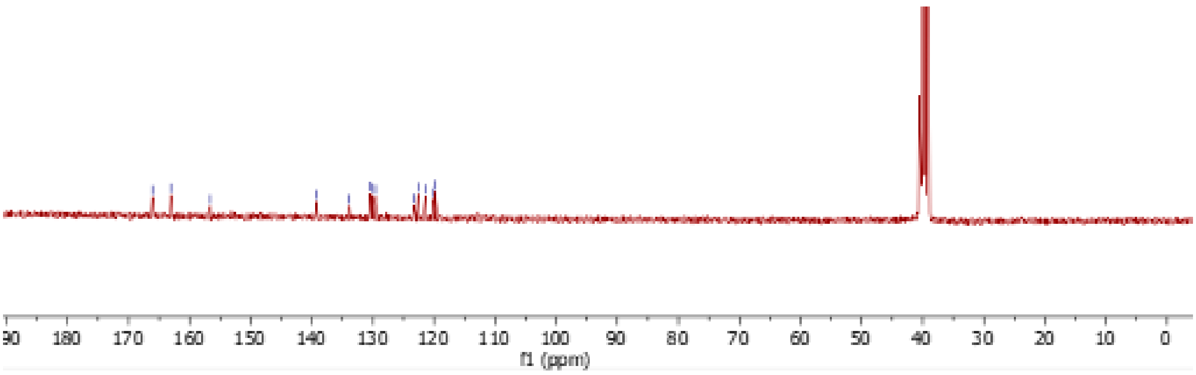

Figure S14 ^{13}C NMR spectrum of compound **6**

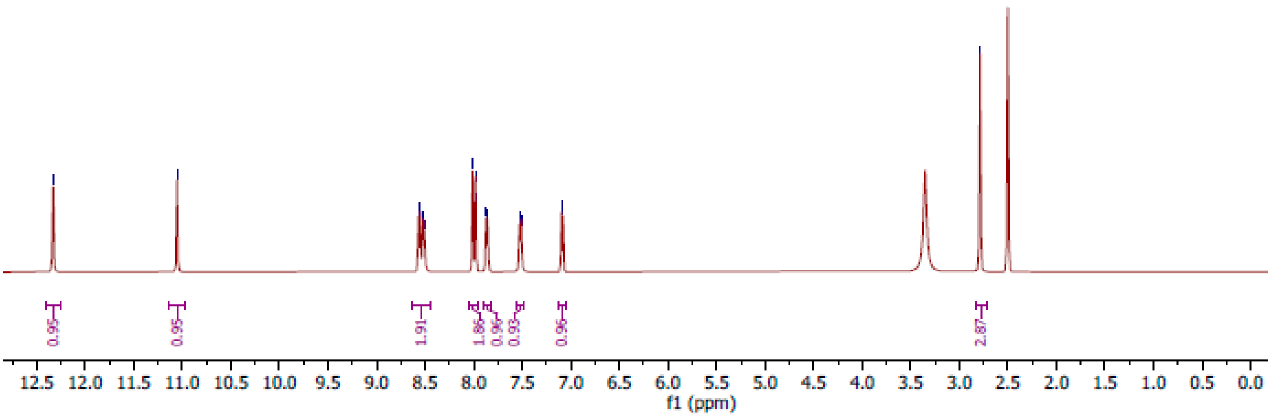

Figure S15 ^{1}H NMR spectrum of compound **7**

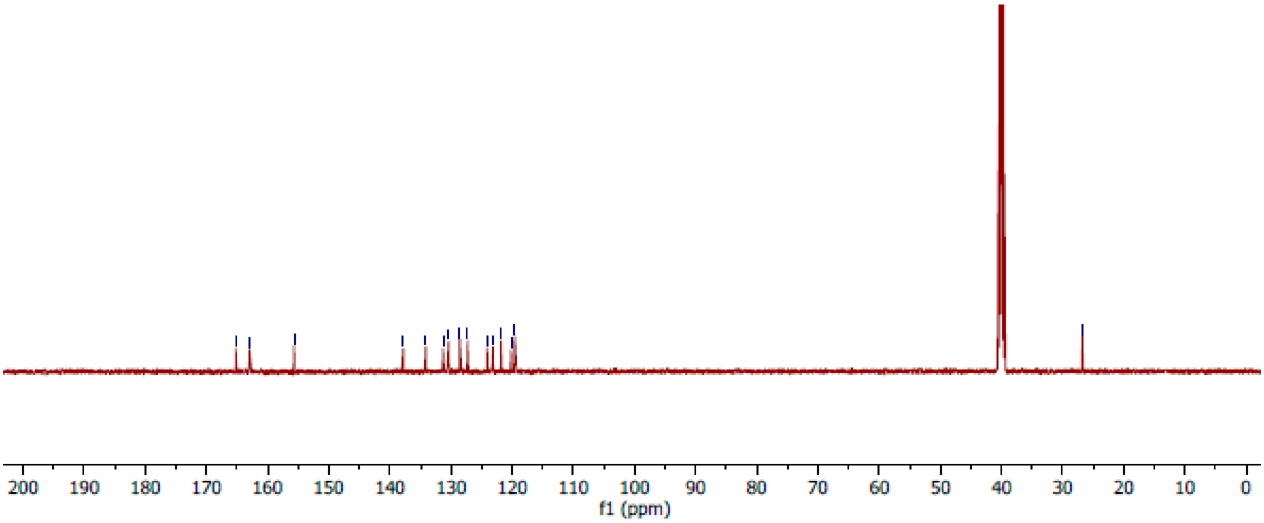

Figure S16 ^{1}H NMR spectrum of compound **7**

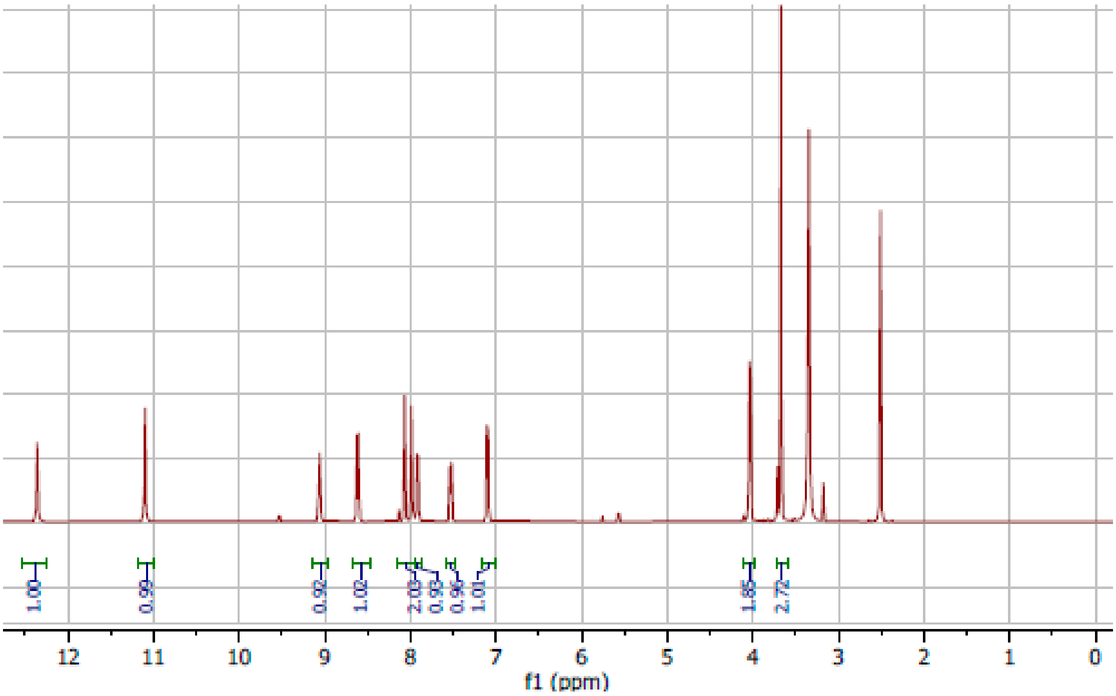

Figure S17 ^{1}H NMR spectrum of compound **8**

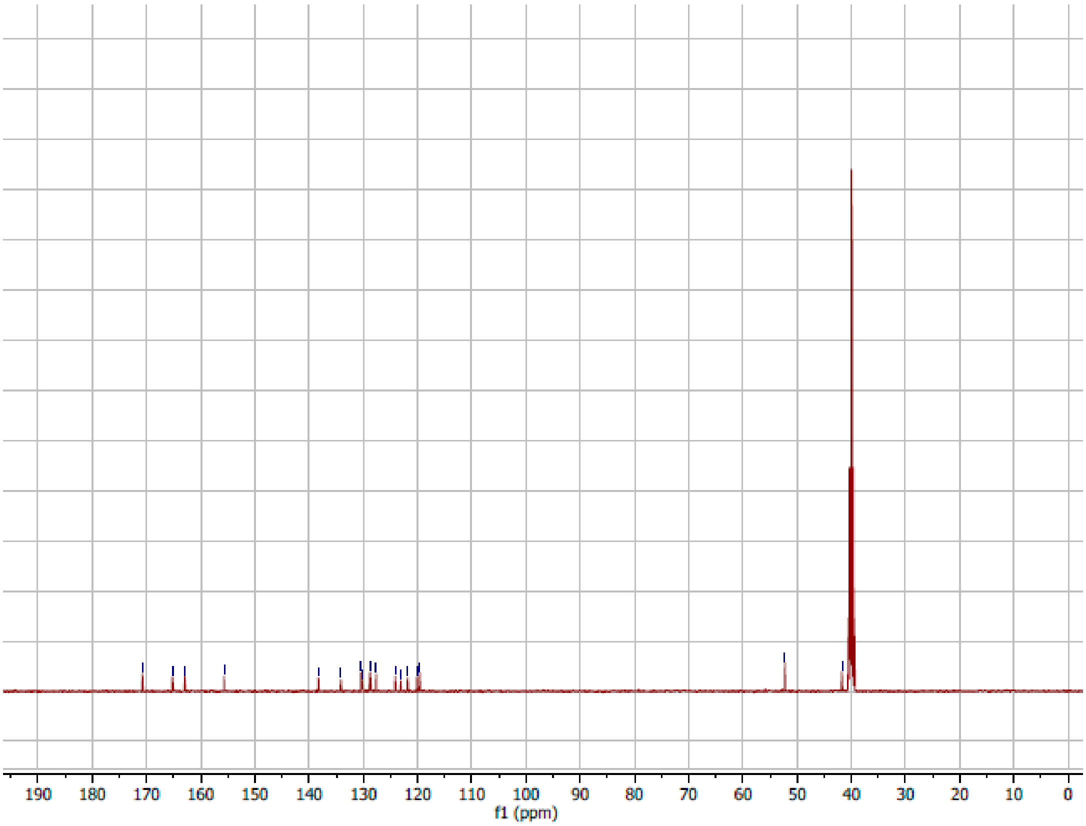

Figure S18 ^{13}C NMR spectrum of compound **8**

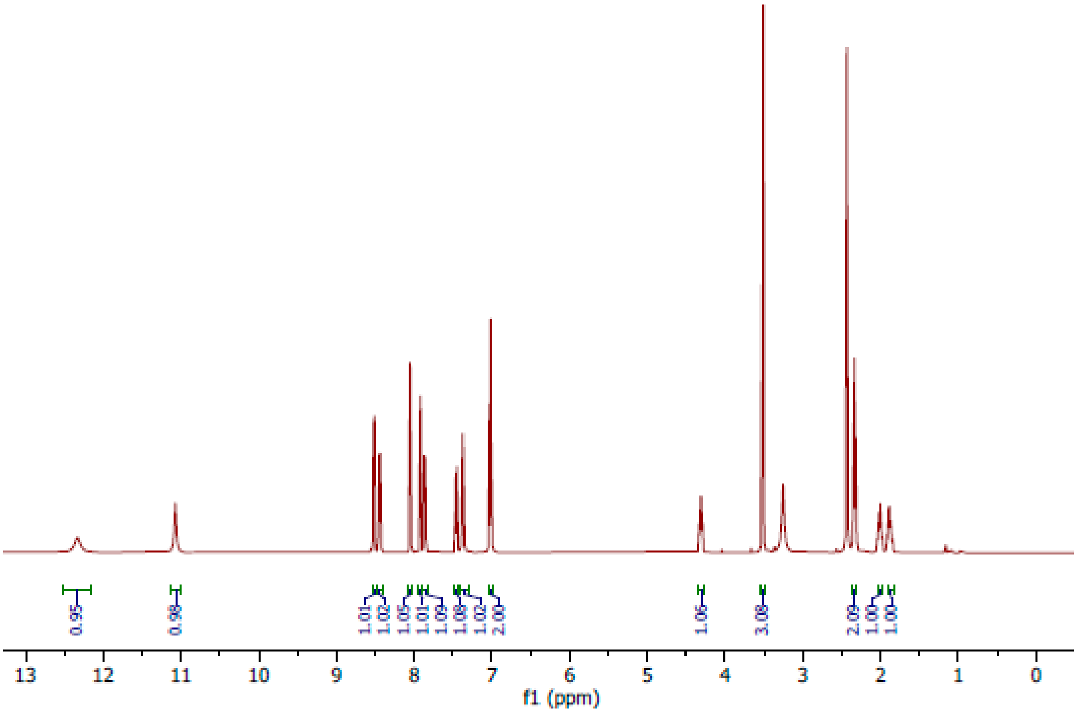

Figure S19 ^{1}H NMR spectrum of compound **9**

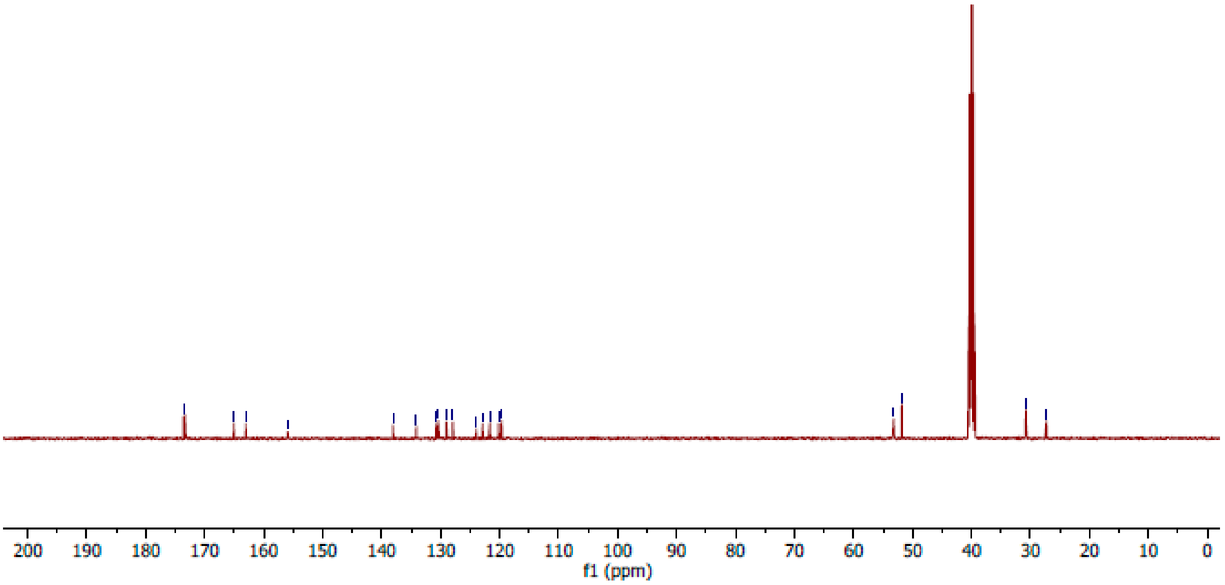

Figure S20 ^{13}C NMR spectrum of compound **9**

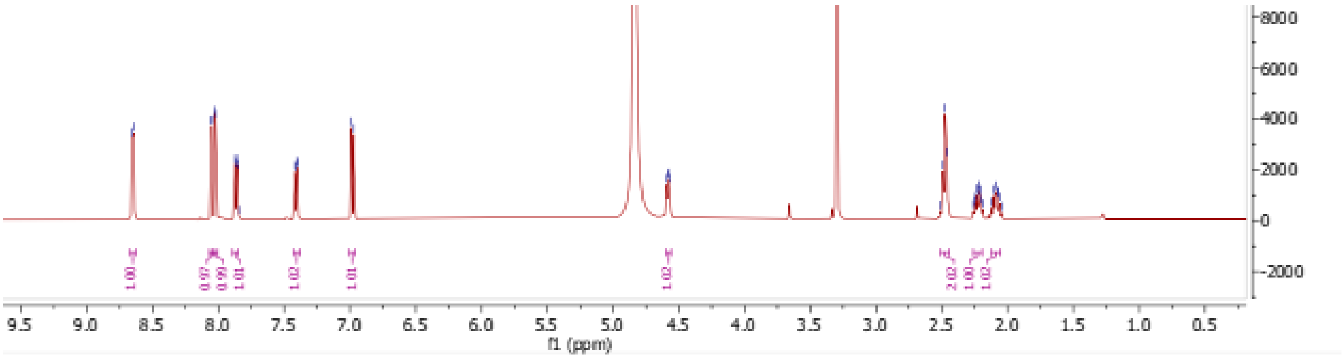

Figure S21 ^{1}H NMR spectrum of compound **10**

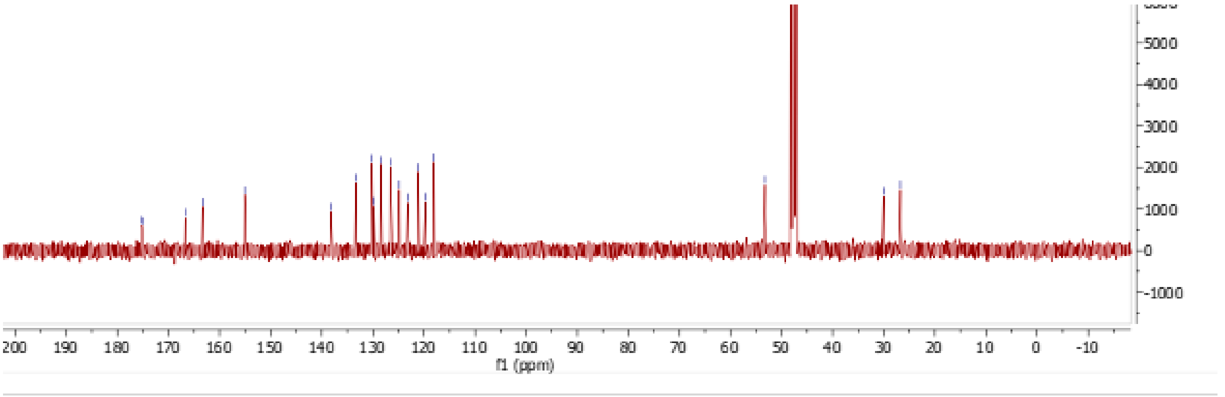

Figure S22 ^{13}C NMR spectrum of compound **10**

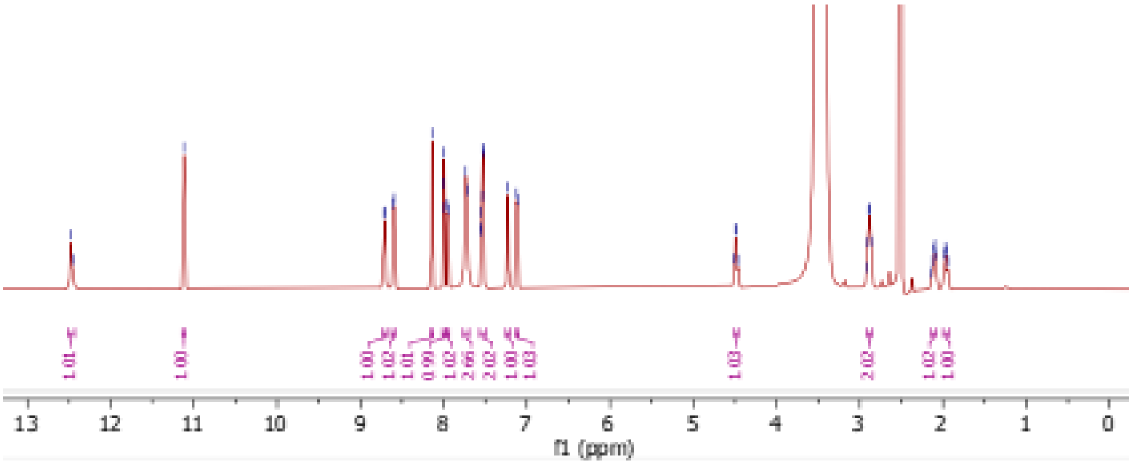

Figure S23 ^{1}H NMR spectrum of compound **11**

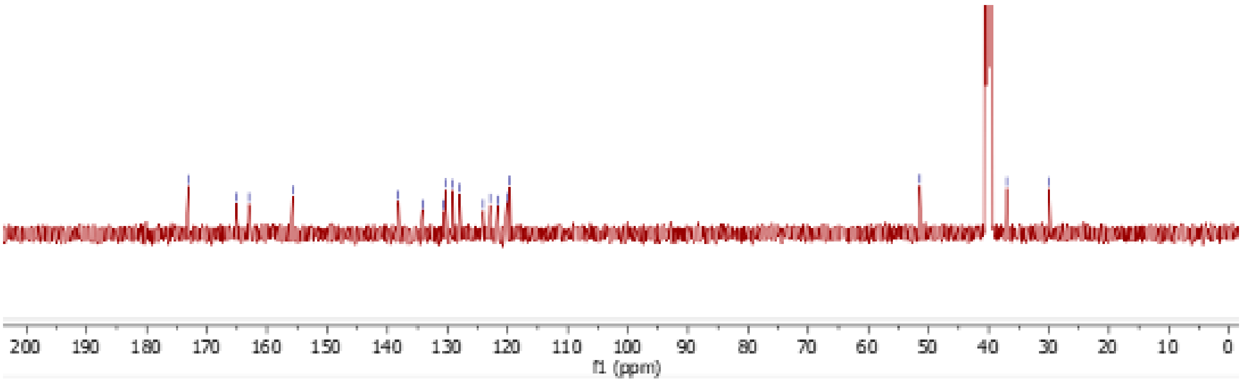

Figure S24 ^{13}C NMR spectrum of compound **11**

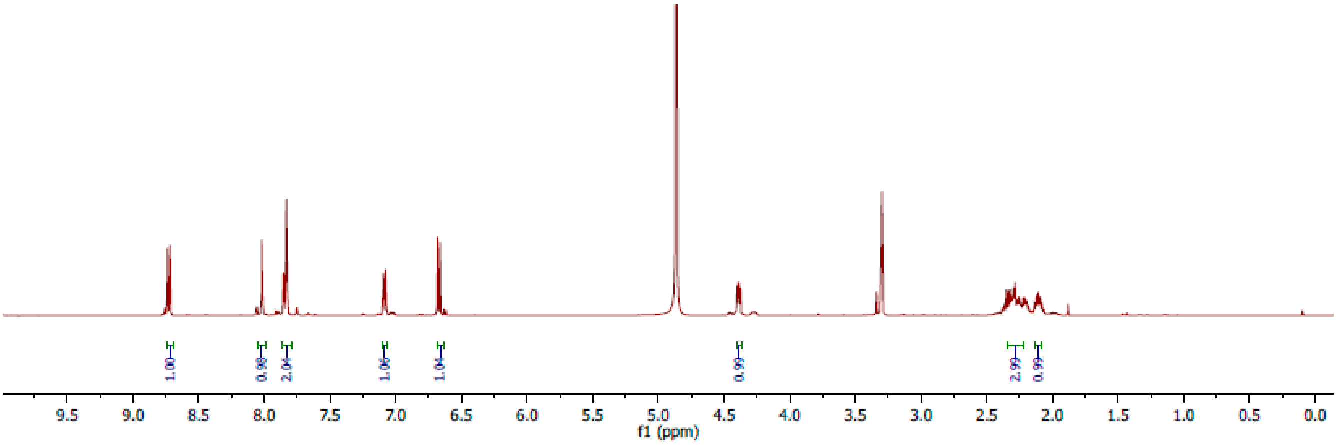

Figure S25 ^{1}H NMR spectrum of compound **12**

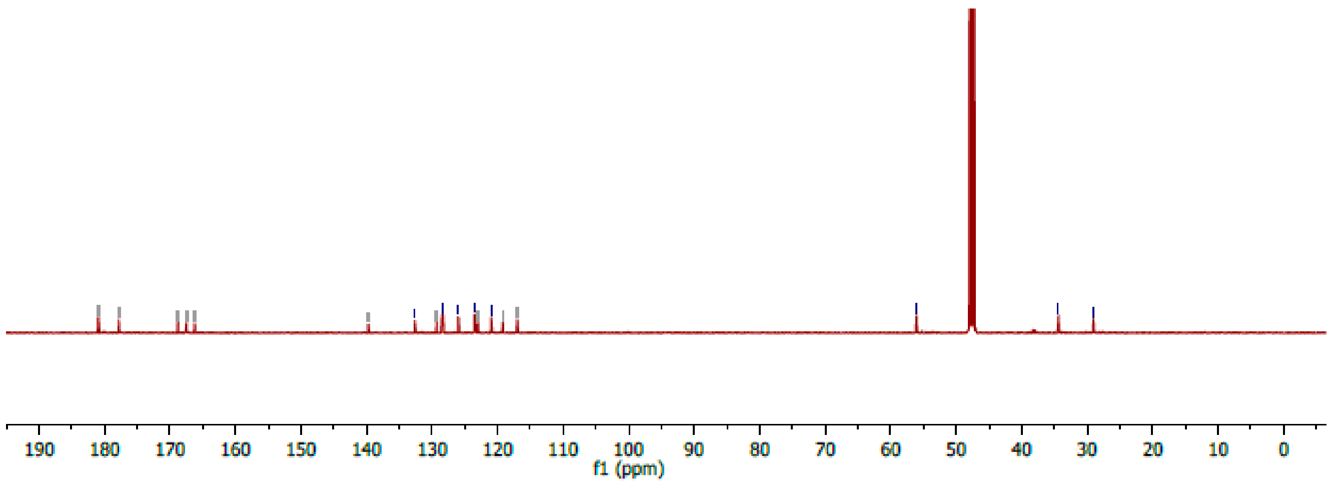

Figure S26 ^{13}C NMR spectrum of compound **12**

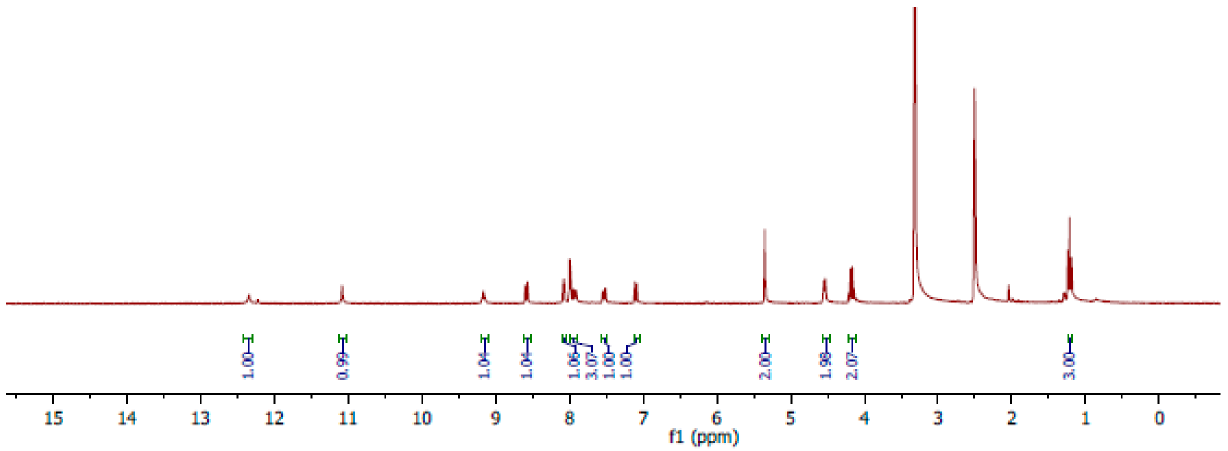

Figure S27 ^{1}H NMR spectrum of compound **13**

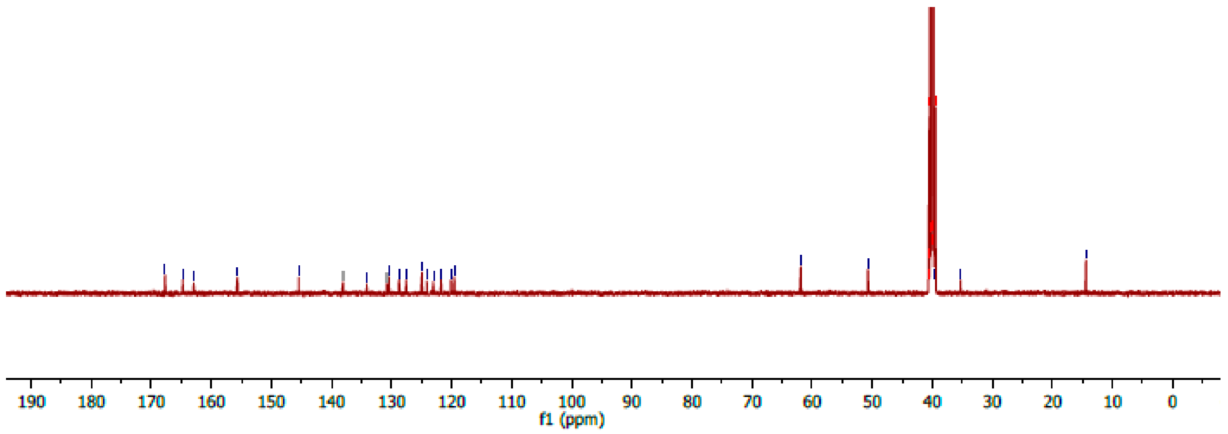

Figure S28 ^{13}C NMR spectrum of compound **13**

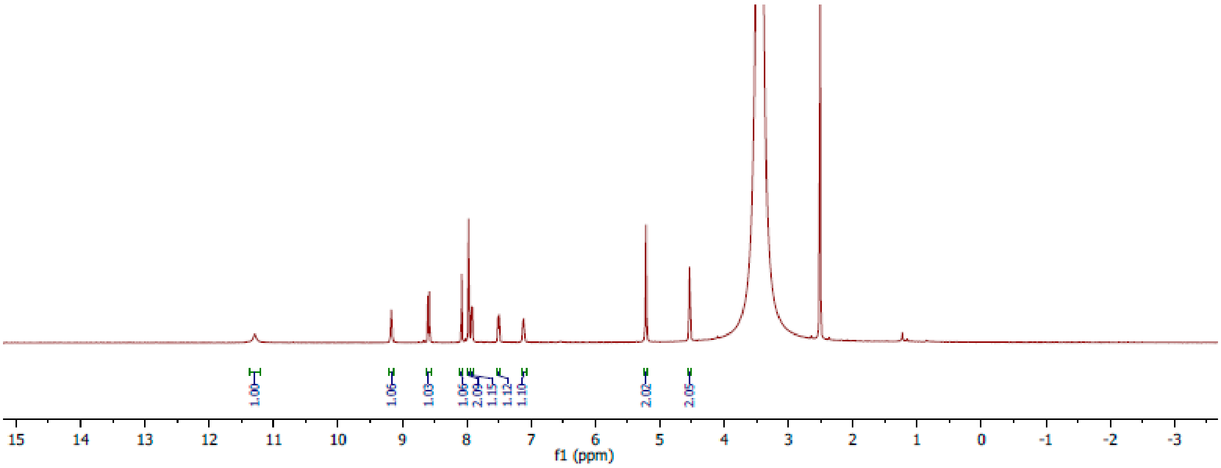

Figure S29 ^{1}H NMR spectrum of compound **14**

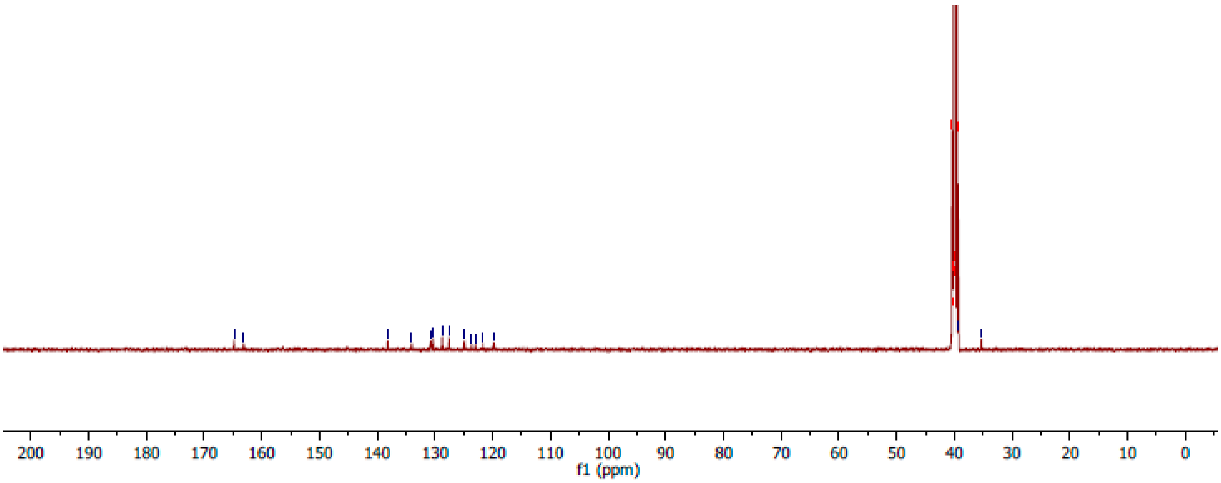

Figure S30 ^{13}C NMR spectrum of compound **14**

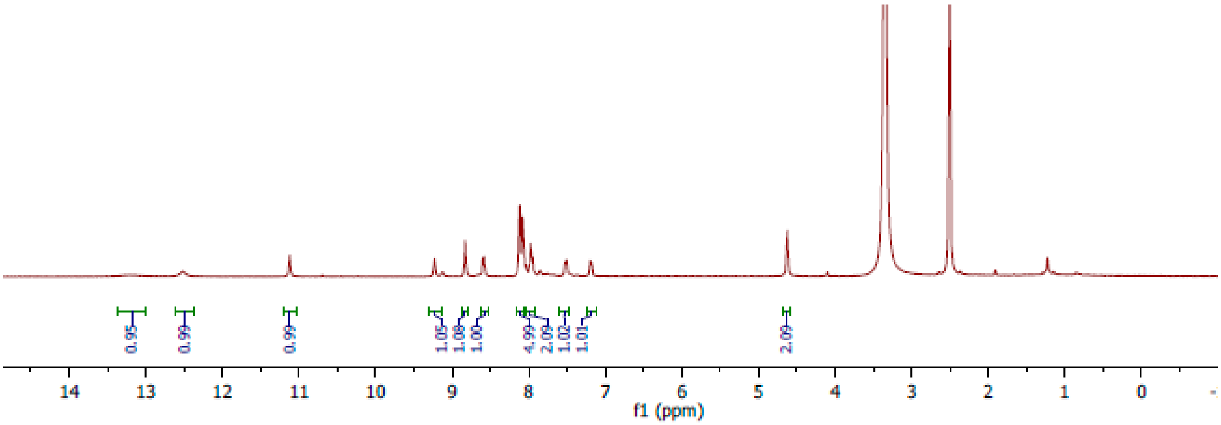

Figure S31 ^{1}H NMR spectrum of compound **15**

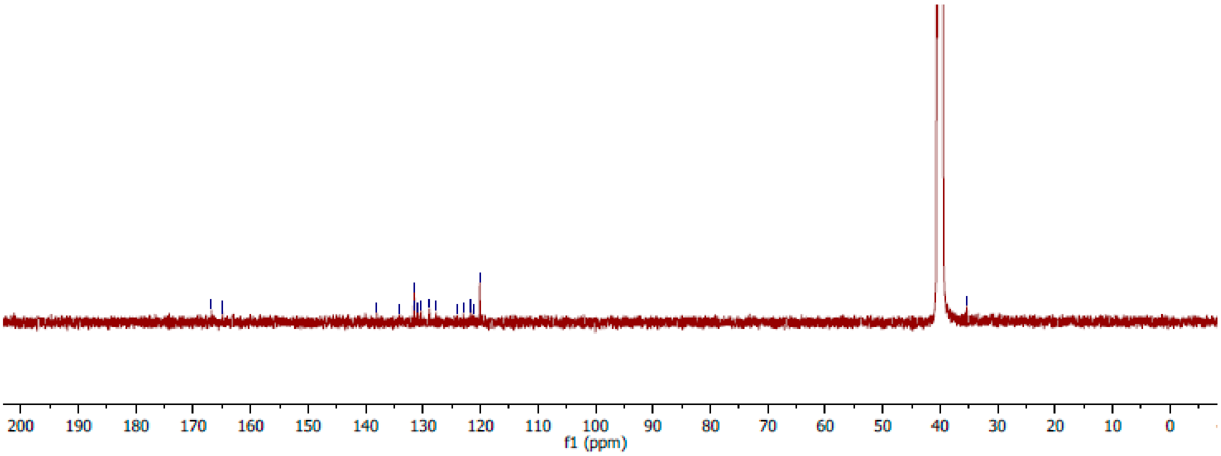

Figure S32 ^{13}C NMR spectrum of compound **15**

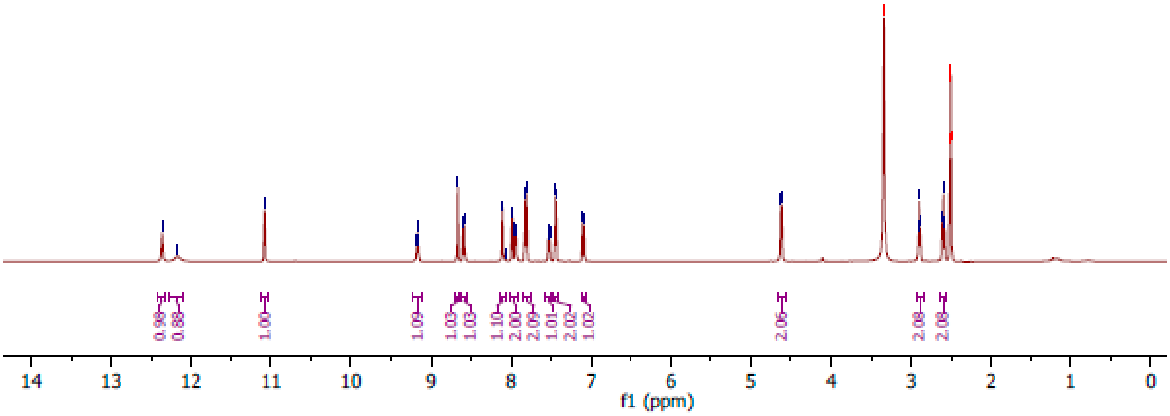

Figure S33 ^{1}H NMR spectrum of compound **17**

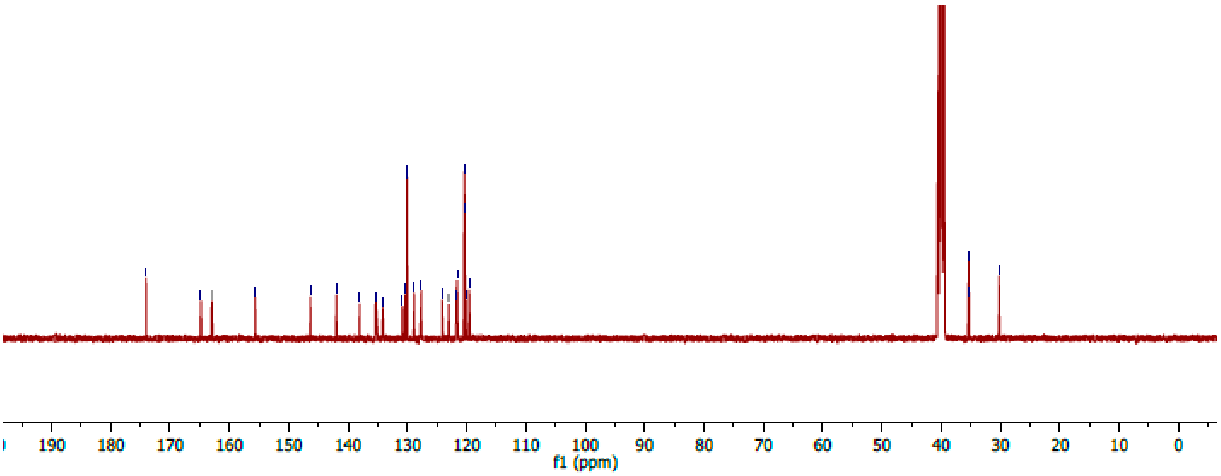

Figure S34 ^{13}C NMR spectrum of compound **17**

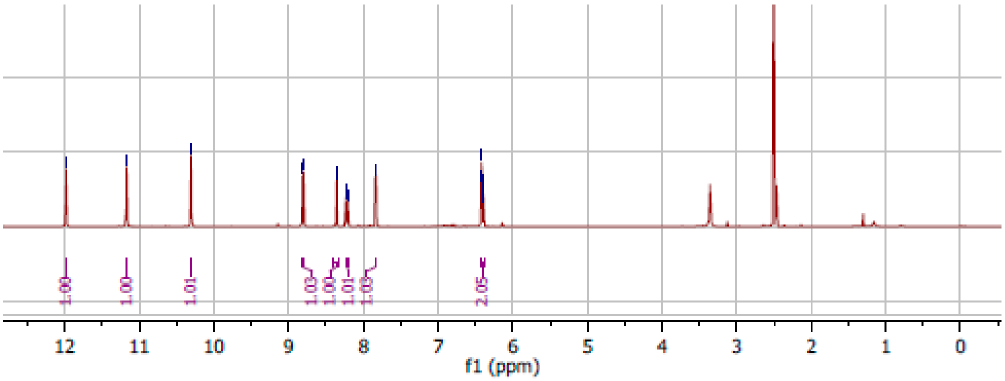

Figure S35 ^{1}H NMR spectrum of compound **18**

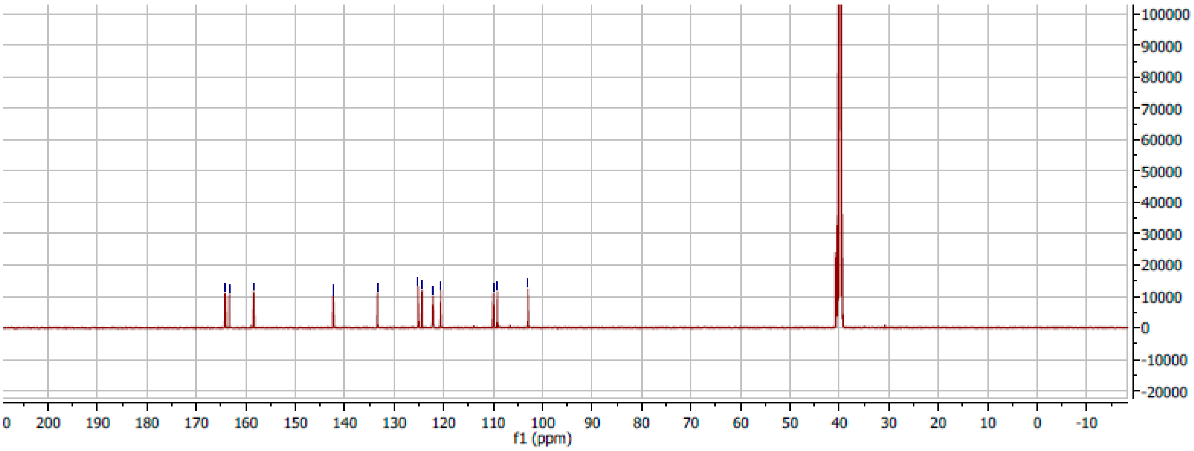

Figure S36 ^{13}C NMR spectrum of compound **18**

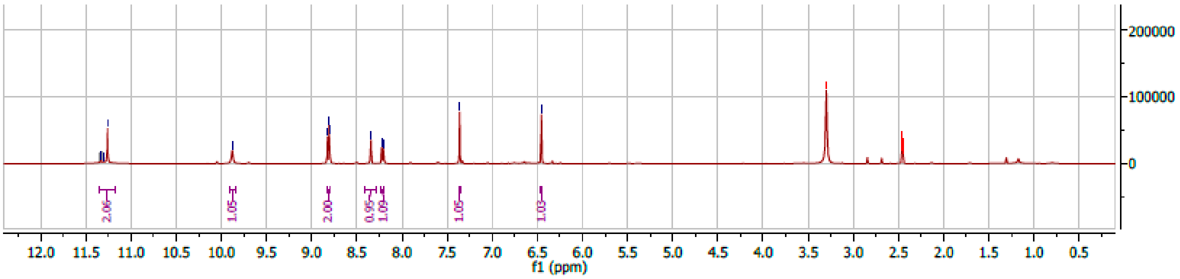

Figure S37 ^{1}H NMR spectrum of compound **19**

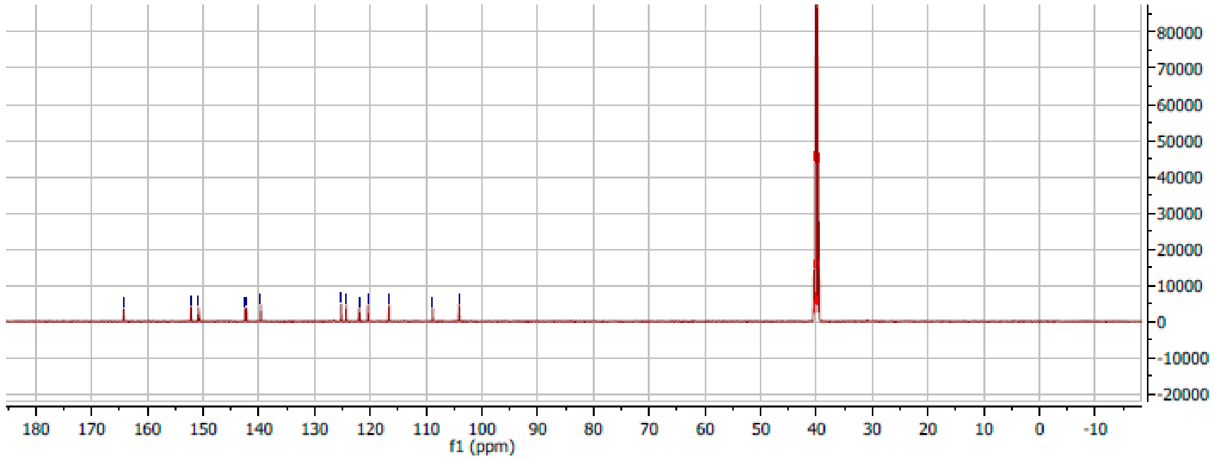

Figure S38 ^{13}C NMR spectrum of compound **19**

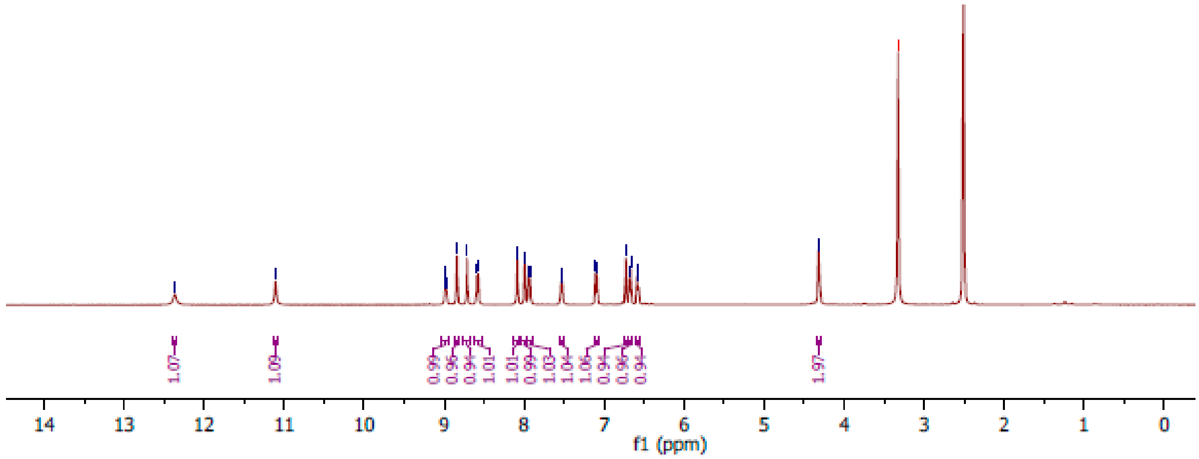

Figure S39 ^{1}H NMR spectrum of compound **20**

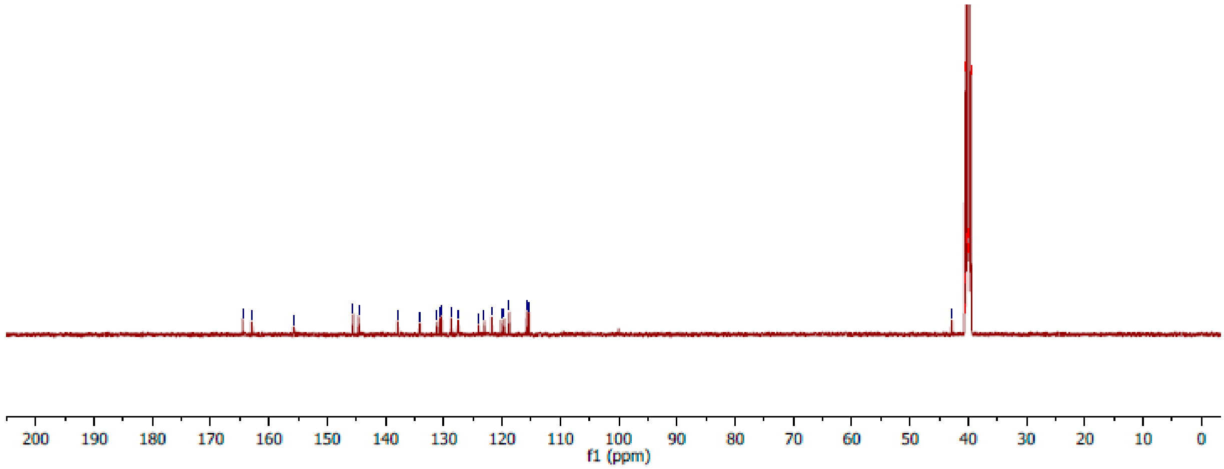

Figure S40 ^{13}C NMR spectrum of compound **20**

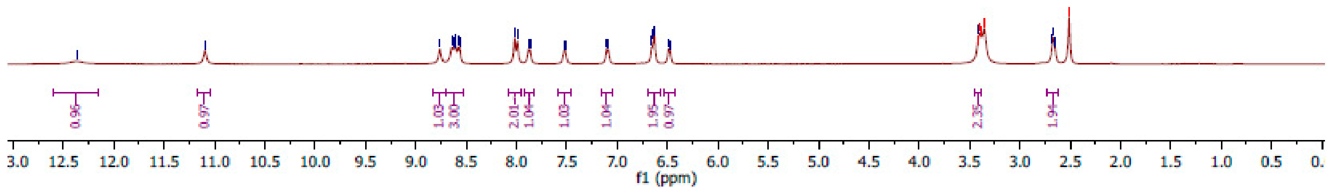

Figure S41 ^{1}H NMR spectrum of compound **21**

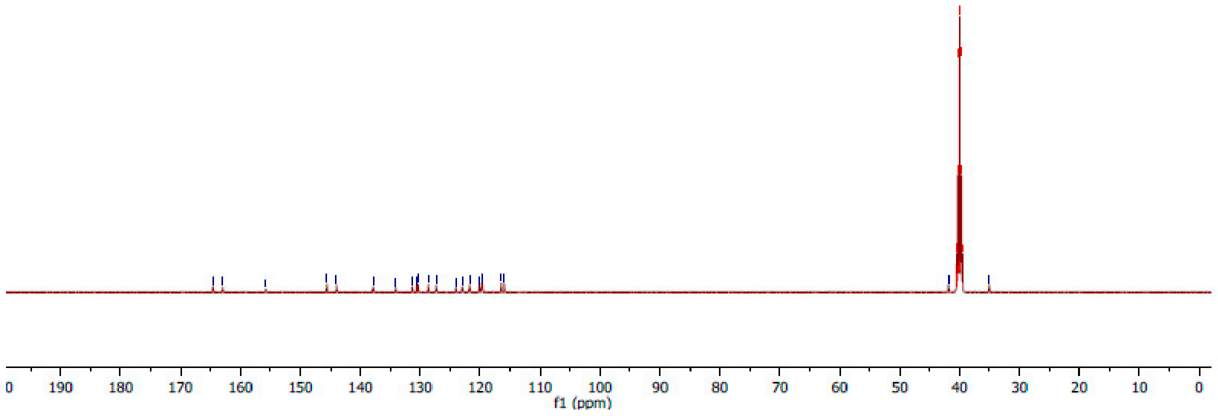

Figure S42 ^{13}C NMR spectrum of compound **21**

Extended Biological Data

Table S1 Minimum inhibitory concentration (MIC) in µg/mL of compounds **1-21** against 4 colistin-resistant strains of GNB. KP = *Klebsiella pneumoniae,* EC = *Escherichia coli.*

Compound	KP113250	KP113254	EC94393	EC94474
**1**	>128	>128	>128	>128
**2**	>128	>128	>128	>128
**3**	>128	>128	>128	>128
**4**	>128	>128	>128	>128
**5a**	>128	>128	>128	>128
**5b**	>128	>128	>128	>128
**6**	>128	>128	>128	>128
**7**	>128	>128	>128	>128

**8**	>128	>128	>128	>128
**9**	>128	>128	>128	>128
**10**	>128	>128	>128	>128
**11**	>128	>128	>128	>128
**12**	>128	>128	>128	>128
**14**	>128	>128	>128	>128
**15**	>128	>128	>128	>128
**16**	>128	>128	>128	>128
**17**	>128	>128	>128	>128
**18**	>128	>128	>128	>128
**19**	>128	>128	>128	>128
**20**	>128	>128	>128	>128
**21**	>128	>128	>128	>128

Table S2. Colistin minimum inhibitory concentration (MIC) in combination with 4 µM of compound **5b** and niclosamide against 4 colistin-resistant strains of GNB. KP = *Klebsiella pneumoniae,* EC = *Escherichia coli*.

	Colistin MIC with 4 µM compound (µg/mL)			
**Compound**	**KP113250**	**KP113254**	**EC94393**	**EC94474**
**Niclosamide**	0.25	0.5	0.25	0.5
**5b**	256	256	8	16
**Colistin alone**	256	256	8	16
